# Self-assembly of new cobalt complexes based on [Co (SCN)_4_], synthesis, empirical, antioxidant activity, and quantum theory investigations

**DOI:** 10.1038/s41598-022-18471-7

**Published:** 2022-09-22

**Authors:** Amal Ferchichi, Jawher Makhlouf, Youness El Bakri, Kandasamy Saravanan, Arto Valkonen, Heba E. Hashem, Sajjad Ahmad, Wajda Smirani

**Affiliations:** 1 Labortory of Material Chemistry, Faculty of Sciences of Bizerte, University of Carthage, Bizerte Zarzouna, Tunisia; 2 Department of Theoretical and Applied Chemistry, South Ural State University, Lenin Prospect 76, Chelyabinsk, Russian Federation 454080; 3 Faculty of Chemistry, University of Warsaw, Warsaw, Poland; 4 Department of Chemistry, University of Jyvaskyla, 40014 Jyvaskyla, Finland; 5 Department of Chemistry, Faculty of Women, Ain Shams University, Cairo, Egypt; 6 Department of Health and Biological Sciences, Abasyn University, Peshawar, 25000 Pakistan

**Keywords:** Drug discovery, Chemistry, Mathematics and computing, Physics

## Abstract

The cobalt (II) complexes have been synthesized from the reaction of the cationic entities (3,4-dimethylaniline (**1**) and histamine (**2**)) with metallic salt CoCl_2_⋅6H_2_O and thiocyanate ion (SCN^−^) as a ligand in H_2_O/ethanolic solution and processing by the evaporation crystal growth method at room temperature to get crystals. The synthesized complex has been fully characterized by single-crystal X-ray diffraction. UV–Visible, FTIR spectroscopy, TGA analysis, and DFT circulations were also performed. The crystal structural analysis reveals that the solid (**1**) {[Co(SCN)_4_] (C_8_H_12_N)_3_}·Cl crystallizes in the monoclinic system with the space group P2_1_/n and the solid (2) {[Co(SCN)_4_](C_5_H_11_N_3_)_2_}·2Cl crystallizes in the monoclinic space group P2_1_/m. Metal cations are joined into corrugated chains parallel to the *b*-axis direction in (**1**) and (**2**) by four thiocyanate anions. The crystal structures of (**1**) and (**2**) were calculated using XRPD data, indicating that they are closely connected to the DRX mono-crystal results. Different interactions pack the system into a ring formed by N–H⋯Cl and N–H⋯S hydrogen bonds. C–H⋯π and the π⋯π stacking of anilinuim ring for (**1**) and N–H⋯S intermolecular interactions for (**1**) and (**2**) increase the crystals' robustness. Hirshfeld surface analysis cum 2D fingerprint plots visualize the main intermolecular interactions with their contributions in the solid-state phase. The molecular geometries of both complexes obtained from the crystal structure were used for quantum chemical calculation. Here, frontier orbital analysis and electrostatic potential illustrate the chemical reactivities of metal–organic complexes. QTAIM and NCI analysis reveal the strength of interactions at the electronic level.

## Introduction

The preparation of coordination products depends on cobalt thiocyanate and additional N-donor ligands and has piqued our curiosity for a long time. Several research studies during the last few years have proved the usefulness of transition metal complexes in different disciplines, while Certain transition metal complexes have a long and storied history of usage as antibacterial and antiviral medications. For instance, Co is used to treat herpes, perhaps by preventing the viral DNA polymerase from doing its job. Early transition-metal polyoxoanions, such as antifungal^1,2^, antibacterial^1,3^ antitumor activities^4,5^. Significant progress has been made in the use of transition metal complexes as drugs to treat a variety of human diseases such as carcinomas, antitumor, lymphomas, infection control, anti-inflammatory, diabetes, and neurological disorders properties; pharmacology to cure various illnesses^6,7^; and catalysis for reaction selectivity^8,9^. Since there are several natural products containing nitrogen atom compounds with great medicinal importance^6,10^, research has been focused on the preparation of transition metal complexes with hetero-atoms chelating ligands in our case the amin entity^11–13^.

In most organometallic compounds, the Co(II) cations with a d9 configuration are present in square planar, square-pyramidal, or square-bipyramidal geometries. As a result of the unique reactivity shown by the formed complexes and the type of ligands that control the features of those complexes, the chemistry of cobalt complexes gains great interest in many inorganic chemistry groups. It is also generally known that when some organic molecules (drugs) are supplied in conjunction with metals, they are substantially more effective^14–16^. Furthermore, aniline binding in organometallic complexes shows low-energy delocalized π*-orbitals, which increases the probability of altering optical, physicochemical, electrochemical properties, and structural traits. The aniline derivatives such as 3,5-dimethylaniline add to the 2-(3*H*-imidazol-4-yl) ethanolamine known as histamine which has an important role in several pharmacological processes. These entities have been investigated, and they have essentially provided access to mono and bi-metallic complexes^17–22^. Some of these complexes' biological action was investigated and proved that the ligand's effectiveness increases with its binding position in the metal's coordination sphere. Lately, we have published the synthesis of transition metal complexes with pseudo thiocyanic anions (SCN^−^) as ligands^23–27^. To define the intermolecular interactions and illustrate the crystalline configuration, the Hirshfeld surface analysis, and various spectroscopic studies were carried out to characterize the complexes.

## Experimental

### Chemical preparation

All of the chemicals were utilized without being purified. The products were obtained by mixing at ambient temperature.

To 25 mL of an aqueous solution of CoCl_2_ anhydrous, 0.6 g of 3,4-dimethylaniline dissolved in 25 mL of diluted chloric acid was added. Under continuous stirring, a 25 mL aqueous solution of KSCN was carefully added. At room temperature, the finished solution slowly evaporated. After a week, a single-crystal X-ray structural analysis revealed a blue crystal. The solid metal complex was obtained by stirring together the organic ligand solution with the 2-(3*H*-imidazol-4-yl)ethanolamine known as histamine and CoCl_2_·6H_2_O. The thiocyanic acid solution (HSCN), which is produced from a cationic resin, was introduced dropwise to the well-stirred blue mixture to exchange H-SO_3_ with KSCN. At ambient temperature, the resulting combination was allowed to evaporate for a week. After 8 days, dark blue crystals suitable for single-crystal X-ray structure analysis were afforded.

### Investigation techniques

#### X-ray single-crystal structural analysis

A suitable single crystal of (**1**) and (**2**) was carefully chosen under a polarizing microscope for X-ray diffraction structural investigation. Data were collected at 170 K using graphite-monochromated Mo *K*α radiation on a Bruker-Nonius Kappa CCD with an APEX II detector diffractometer (λ = 0.71073 Å). The structures were solved using the SHELX program's dual space method, then refined using successive differential Fourier syntheses and a full-matrix least-squares procedure using the SHELXL program^28,29^. The drawings were made with Diamond^30^. Table 1 shows the crystal data and experimental conditions utilized to collect intensity data.

**Table 1:** Crystallographic Data for {[Co(SCN)_4_] (C_8_H_12_N)_3_}·Cl and {[Co(SCN)_4_] (C_5_ H_11_ N_3_)_3_]·2Cl.

Formula	{[Co(SCN)_4_] (C_8_H_12_N)3}·Cl	{[Co(SCN)_4_] (C_5_ H_11_ N_3_}·2Cl
System	Monoclinic	Monoclinic
Space group	P 2_1_/n	P 2_1_/m
**Unit cell dimensions**		
a, b, c (Å)	11.5555 (5), 28.1128 (11), 11.6654 (3)	5.5748 (2), 24.3096 (4), 9.3531 (2)
β (°)	113.480 (2)	99.765 (1)
V (Å^3^)	3475.8 (2)	1249.18 (6)
Mr (g/mol)	693.26	588.48
Dx (Mg m^−3^)	1.325	1.565
Z	4	2
T (K)	170	170
θmax, θmin (°)	28.4, 2.0	27.9, 0.4
µ (mm^−1^)	0.84	1.26
Shape, color	Prism, blue	Long plate, blue
Crystal size (mm^3^)	0.29 × 0.21 × 0.14	0.28 × 0.20 × 0.05
Tmax, Tmin	0.746, 0.642	0.746, 0.664
Diffractometer	Bruker–Nonius Kappa CCD	Bruker–Nonius Kappa CCD
Measured reflections	15,881	2314
independent reflections	8562	1830
F (000)	1444	602
h	− 15 .. 15	− 6 .. 6
k	− 37 .. 37	29 .. −29
l	− 15 .. 15	0 .. 11
**Parameters refined**		
R[F^2^ > 2σ(F^2^)]	0.076	0.044
wR(F^2^)	0.150	0.085
S	1.02	1.08
δρmax, δρmin (eÅ^−3^)	0.53, − 0.39	0.41, − 0.49

With w = 1/[σ^2^(F_o_^2^) + (0.0391P)^2^ + 4.7727P] where P = (F_0_^2^ + 2F_c_^2^)/3.

#### Powder X-ray diffraction

Powder X-ray Diffraction (PXRD) measurements for hand-ground polycrystalline samples were performed at room temperature on a Miniflex600 Rigaku powder X-ray diffractometer using Cu *K*α radiation (λ = 1.540598 Å). Diffraction data were collected in the angular range 2θ = 0–70° with a scan step width of 0.05° and a fixed time of 0.2 s. Rietveld refinement was applied to model the data sets using the GSAS package incorporated with the EXPGUI interface^31^. As a template, the structure determined from single-crystal XRD was employed. The scale factor, background, lattice parameters, and zero-point were refined until convergence.

#### Thermogravimetry differential thermal analysis (TG-TDA)

The thermal analysis spectra for the titled compounds were acquired with a simultaneous thermogravimetry–differential thermal analysis (TG–DTA) utilizing a PyRIS 1 TGA instrument with 14.9 mg for (**1**) and 12.25 mg for (**2**), for a heating rate of 5 °C min^-1^ in the temperature range [300–880 K] under inert atmosphere (nitrogen gas).

#### Infrared spectroscopy

A spectrometer NICOLET IR 200 FT-IR was used to obtain the Fourier Transform Infrared (FTIR) spectrum of a powder sample of the chemicals. 4000–400 cm^−1^ was the scanning range.

#### UV–Visible spectroscopy

A Perkin Elmer Lambda spectrophotometer was used to make the UV measurements. Scans were performed in the 200–800 cm^−1^ range.

#### Antioxidant activity

The anti-cancer medication cisplatin, which is based on platinum, has boosted the usage of metal-containing products in medicine^32^. Helicobacter pylori infections and peptic ulcers are the most common uses for cobalt compounds^33^. To combat drug resistance, a new technique involving the construction of compounds based on the incorporation of bioactive molecules has recently emerged as an appealing strategy. Anilines, for instance, are pharmacophore entities that play a key role in several marketed medications, including the Merck HIV protease inhibitor Crixivan and others^34^. Furthermore, their compounds have excellent biological features, such as anticancer activity in prostate cancer treatment^35^ antimalarial^36^, and antiarrhythmic^37^.

#### DPPH radical scavenging activity

Barca and al.^38^ described a DPPH radical scavenging assay that worked. The DPPH solution (35 µg/L) was diluted with various dilutions of the methanolic solution of the studied compounds and the standard compound (ascorbic acid) (0.25–1 mg/mL). With methanol as the blank, the mixture was placed in the dark for 30 min before monitoring the absorbance at 517 nm (until steady absorbance values were achieved). All of the tests were done in triplicate, and the results were expressed as the mean standard deviation (SD), with ascorbic acid as the reference. The following equation was used to compute the inhibitory percentages of the produced material:

$$\% {\text{Inhibition}} \,  {\text{of}} \, {\text{ DPPH}} \, {\text{radical}}= [({\text{Abs}} \,  {\text{cont}} - {\text{Abs}}  \, {\text{test}}) /{\text{Abs}} \, {\text{cont}}] \times 100$$

where Abs cont = absorbance of the control (reacting mixture without the test sample) and Abs test = absorbance of reacting mixture with the test sample.

The percentage of scavenging activity was plotted against the sample concentration to calculate the IC50, which is defined as the sample concentration required to cause 50% inhibition.

#### Ferrous ion chelating (FIC) ability

Singh and Rajini^39^ developed a method for determining FIC ability. To FeSO_4_ (0.1 mM) and ferrozine, a methanolic solution of the investigated compounds was added at varied concentrations (from 0.25 to 1 mg/mL) (0.25 mM). The tubes were thoroughly shaken before being set aside for 30 min. At 565 nm, the absorbance was measured. The formula was used to calculate each sample's ability to chelate ferrous ions:

 % FIC = [(Acont – Atest)/Acont] × 100. The results were expressed as IC50.

#### Ferrous reducing power

The new compound's reducing power was determined using the method provided by Pulido et al.^40^. With 2.5 mL of phosphate buffer (0.2 M) and 2.5 mL of 1% potassium ferricyanide, a methanolic solution of the chemical (1 mL) at various concentrations (between 0.25 and 1 mg/mL) was produced and incubated at 50 °C for 20 min. This mixture was calculated by centrifuging at 3000 rpm for 20 min after adding 2.5 mL of 10% trichloroacetic acid. The upper layer (2.5 mL) was made up of 2.5 mL of deionized water and 0.5 mL of ferric chloride (0.1%). The standard ascorbic acid solution was treated in the same way, and the absorbance was measured at 700 nm. The following formula was used to calculate the percentage increase in the reducing ability: (%) = [(Atest-Acontrol)/Acontrol] × 100.

The results were also given as an IC50 value.

#### Hirshfeld surface analysis (HSA)

In crystalline materials, the different types of non-covalent interactions are the main aspect to study the crystal packing and arrangement of molecules using HSA and the associated 2D fingerprint plots with the help of Crystal Explorer software 21.5^41^. In general, the Hirshfeld surface map allows for visualization of different features like d_i_, d_e_, d_norm_, shaped index, and electrostatic potential map. The d_norm_ map is called normalized contact distance which is determined by the distances to the closest atom outside (d_e_) and inside (d_i_) surfaces. In the d_norm_ map, three different colors (red, blue, and white) indicate hydrogen bonding, Van der Waals, and interatomic contacts, respectively. To understand the contribution of intermolecular contacts, the enrichment ratio (E) was also calculated; here, the favored contacts are forming while pairing atoms (XY) exhibit a high propensity to form interactions in the crystal packing^42^. In this study, two new Co-metal complexes have been used to perform Hirshfeld surface analysis with the help of a crystallographic information file (CIF).

#### Quantum chemical calculation

By utilizing the Gaussian 09 package program^43^, quantum chemical studies for both Co-metal complexes were performed by B3LYP/LANL2DZ (Los Alamos National Laboratory 2 double) as the level of theory for optimizing geometries of Co-metal atom and 6-311G** basis set for remaining atoms in both complexes^44^. Initial geometries of both metal complexes were attained by the single-crystal x-ray diffraction. Further, the electrostatic potential, molecular orbital analysis, natural bond orbital, and nonlinear optical analysis were performed by utilizing optimized geometries. The optimized structure, frontier molecular orbitals, and ESP maps were visualized using Gauss view^45^ and 3Dplot^46^ software. Further, the global reactivity descriptors (units in eV), such as ionization potential, electron affinity, electronegativity, chemical potential, global hardness, and electrophilicity were calculated with the help of formulae based on Koopmans’ theorem^47–52^.

Noncovalent interactions are playing an important role to determine the shape and supramolecular architecture of crystals in the solid-state phase^53^. The metal complexes are forming different types of noncovalent interactions that significantly influence the crystal structures. In general, the quantum crystallography method helps to understand the nature of intermolecular interactions in crystals at the electronic level beyond geometrical parameters. The wave-function calculation is an alternative way of modeling the diffraction data. In recent years, quantum chemical analysis followed by non-covalent interactions and QTAIM (quantum theory of atoms in molecules) have also shown advanced and gained an enormous amount of interest among researchers due to valuable results behind experiments^54^. The wave function for both complexes was generated from the crystal structure and this was used for non-covalent interaction analysis to get more accurate results than gas phase analysis.

## Results and discussion

### X-ray diffraction analysis

**Figure 1:**
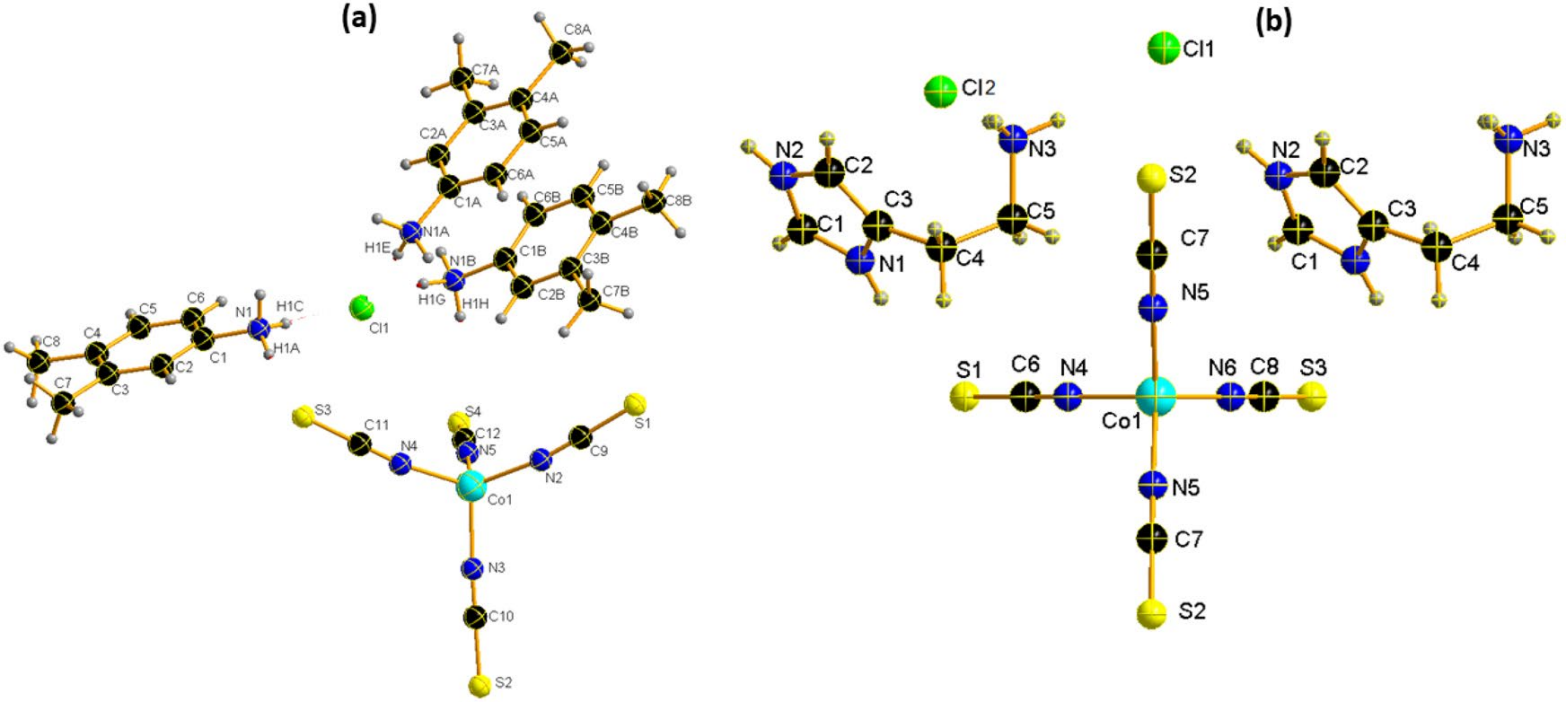
ORTEP Views of (**a**) {[Co(SCN)_4_] (C_8_H_12_N)_3_}·Cl and (**b**) {[Co(SCN)_4_] (C_5_ H_11_ N_3_)_3_}·2Cl.

(1) The blue prismatic crystal of the coordination complex [Co(NCS)_4_] (3,4-dimethylanilinuim)_2_ Cl (**1**) is obtained and crystallizes in a monoclinic space group P2_1_/n with formula units Z = 4 in the unit cell. The asymmetric unit contains one Co (II) cation on a center of inversion with four thiocyanates anion and three 3,4-dimethylanilinuim ligands added to a chloride ion crystallographically independent (Fig. 1a).

**Figure 2:**
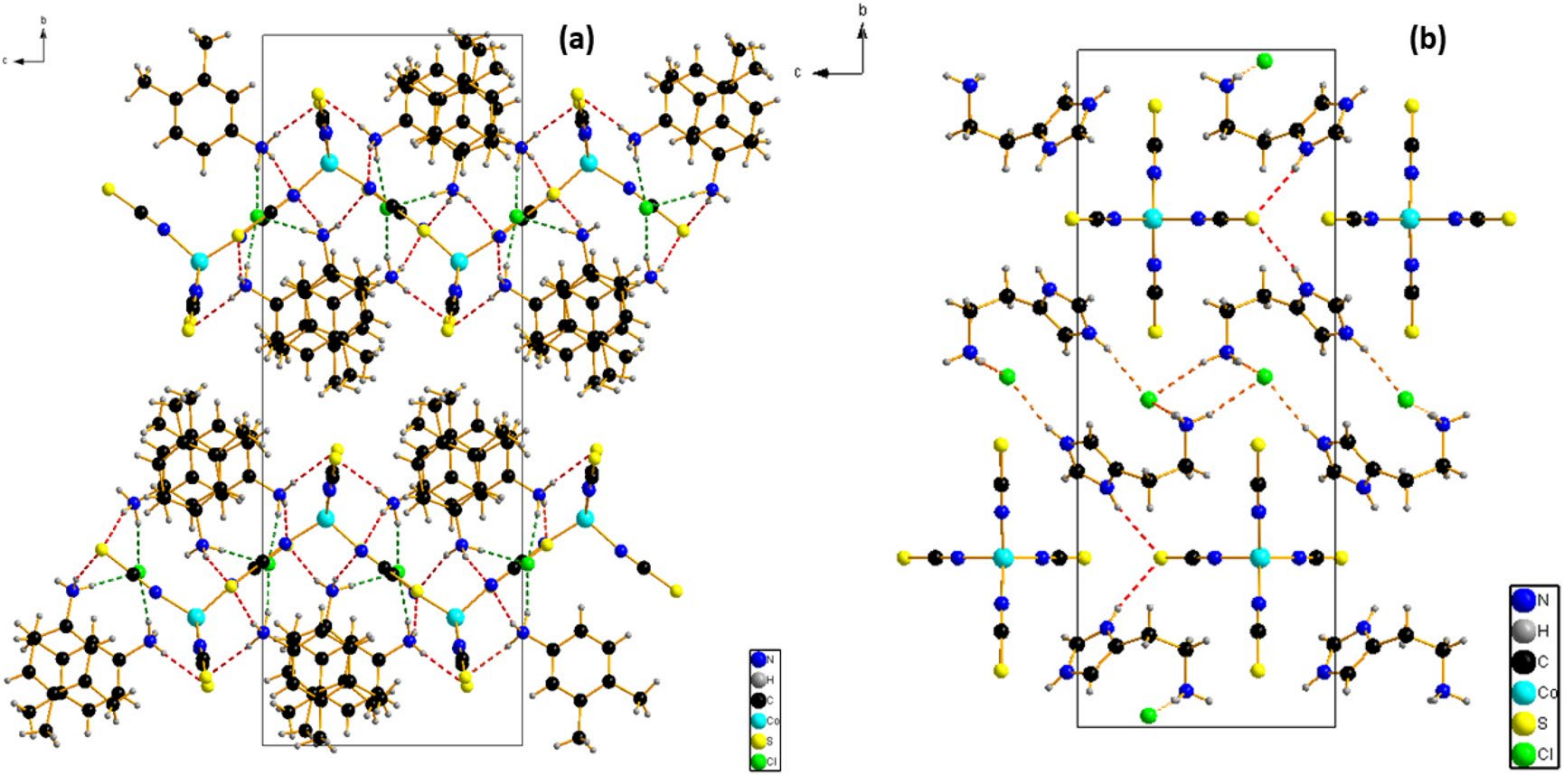
Projection along the *a*-axis of the structure of (**a**) {[Co(SCN)_4_] (C_8_H_12_N)_3_}·Cl and (**b**) {[Co(SCN)_4_] (C_5_ H_11_ N_3_)_3_}·2Cl.

The Co cations are fourfold coordinated by the four terminal N-bonded thiocyanate anions (N2, N3, N4, and N5) to obtain the inorganic entities [Co(SCN)_4_]^2−^, Cl^−^ ions are located in y = 1/4 et y = 3/4 and the cationic entities (Fig. 2a). The bond angle and the bond lengths around the central Co atoms comparable to the previous literature showed that the tetrahedron is slightly distorted similar to C_10_H_26_N_4_ Co (SCN)_4_^55^ and [Ni(SCN)_4_] 2(C_5_H_7_N_2_)^56^ (Table S1).

The discrete complex is linked via intermolecular N–H⋯Cl hydrogen bonds established between the N atoms of the cationic entity and the chloride atoms, and also via N–H⋯S ones between the N atoms of the cationic entity and the thiocyanate S atoms, into layers parallel to the b/c plane (Fig. 2b). These layers are further connected through hydrogen bonds into a 3D network (Fig. 2a). The intermolecular H-bonding interactions link neighboring entities with lengths from 3.259 (4) Å to 3.374 (4) Å for N–H⋯S H-bonds and from 3.076 (4) Å to 3.158 (4) Å for N–H⋯Cl H-bonds. (Table S2), contributing to ${R}_{4}^{2}$ (14) et ${R}_{4}^{2}$ (8) rings as shown in Fig. 4a.

**Figure 3:**
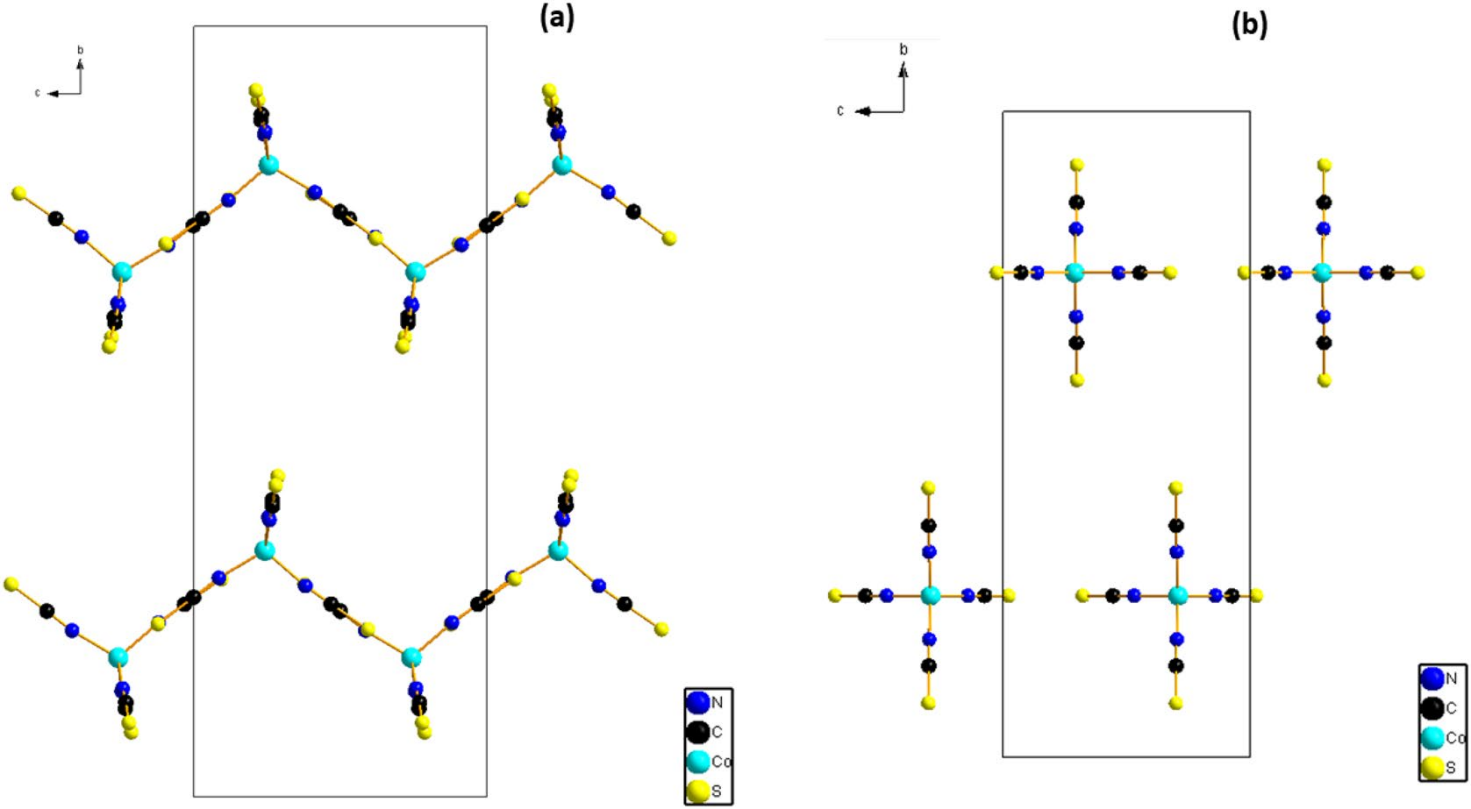
Projection of the anionic part along the *a*-axis of the structure of (**a**) {[Co(SCN)_4_] (C_8_H_12_N)_3_}·Cl and (**b**) {[Co(SCN)_4_] (C_5_ H_11_ N_3_)_3_}·2Cl.

(2) A Long plate blue crystals of coordination polymer {[Co(SCN)_4_] (C_5_H_11_N_3_)_2_}·2Cl are obtained, which crystallizes in a monoclinic system with the P2_1_/m space group. The asymmetric unit comprises one tetra(isothiocyanate) Cobalt [Co(NCS)_4_]^2−^ anions, two chloride ions, and two Histamine cations (Fig. 1b). The bond distances and bond angles are summarized in Table S1. The coordination geometry of the central Co(II) ions in the [Co(NCS)_4_]^2−^ anions, presented as a slightly distorted tetrahedron (Fig. 3b) in which The Co cations are coordinated by N-bonded thiocyanate anions (N4, 2 equivalents of N5 and N6). The Co–N bond distance is 1.949(3) Å and the N–Co–N bond angles in between 115.60 (19)–100.54 (18)°. These values are in agreement with those found in complexes containing the [M(NCS)_4_]^2−^ anion^55–63^.

Figure 3a and b shows that the [Co(NCS)_4_]^2−^ anions are arranged along the a-axis direction. These anions lie at ($\frac{1}{4},$ $\frac{1}{4}$, $\frac{1}{4}$), ($\frac{3}{4},$ $\frac{1}{4}$, $\frac{3}{4}$), to form anionic layers parallel to the (b,c) plane.

**Figure 4:**
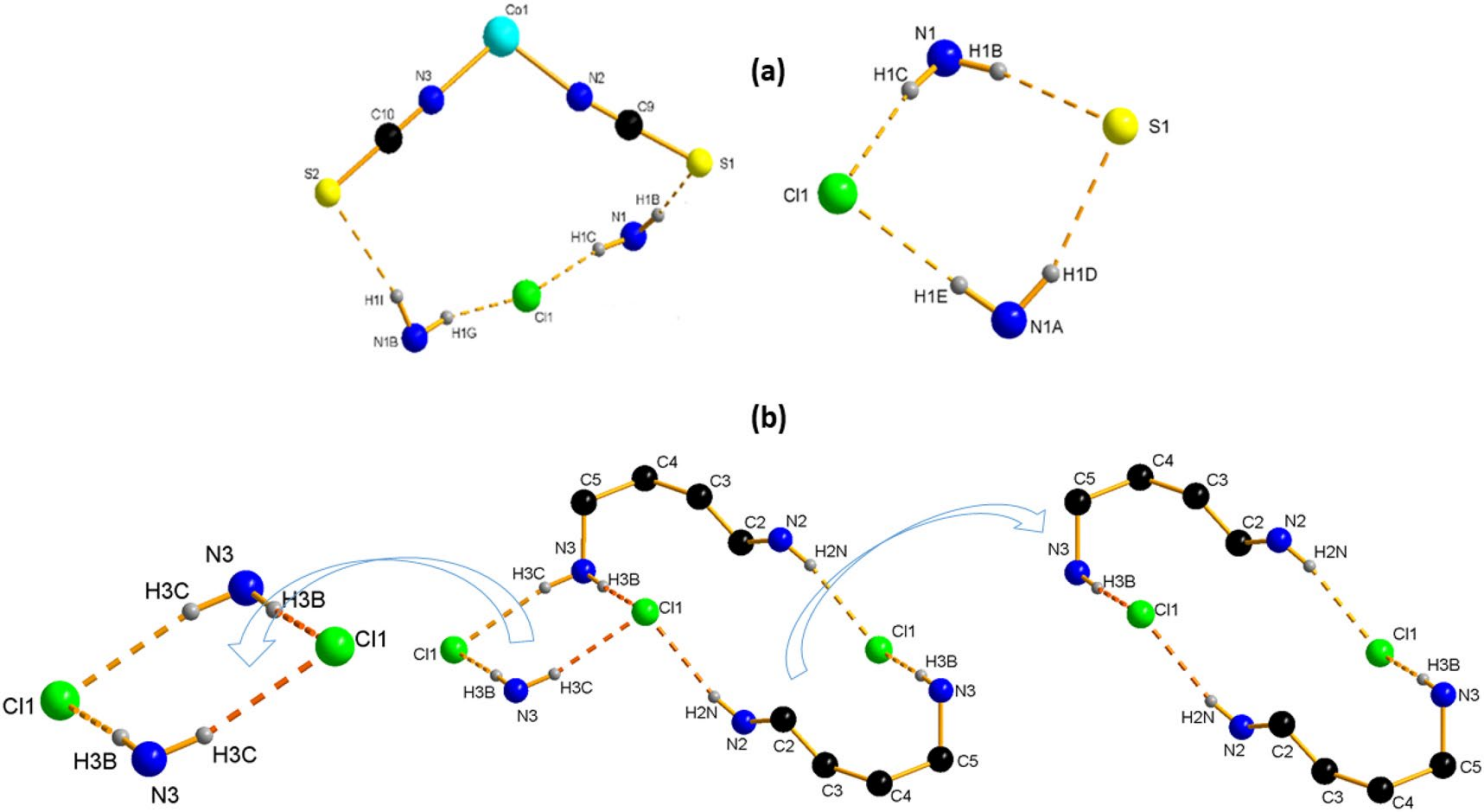
Hydrogen bonds ring (**a**) {[Co(SCN)_4_] (C_8_H_12_N)_3_}·Cl and (**b**) {[Co(SCN)_4_] (C_5_ H_11_ N_3_)_3_}·2Cl.

The interactions between the cations and anions are presented in Fig. 4 as N–H⋯S, and N–H⋯Cl. The network's stability and robustness are improved by these interactions (Table S2). Intermolecular hydrogen bonding interactions connect neighboring thiocyanate anions via N–H⋯S hydrogen bonds with values of 2.48 (2) Å and N–H⋯Cl ones with lengths ranging from 2.30 (2) to 2.32 (2) Å, resulting in the ${R}_{4}^{1}$(8) and t ${R}_{4}^{1}$(18) rings depicted in Fig. 4b.

**Figure 5:**
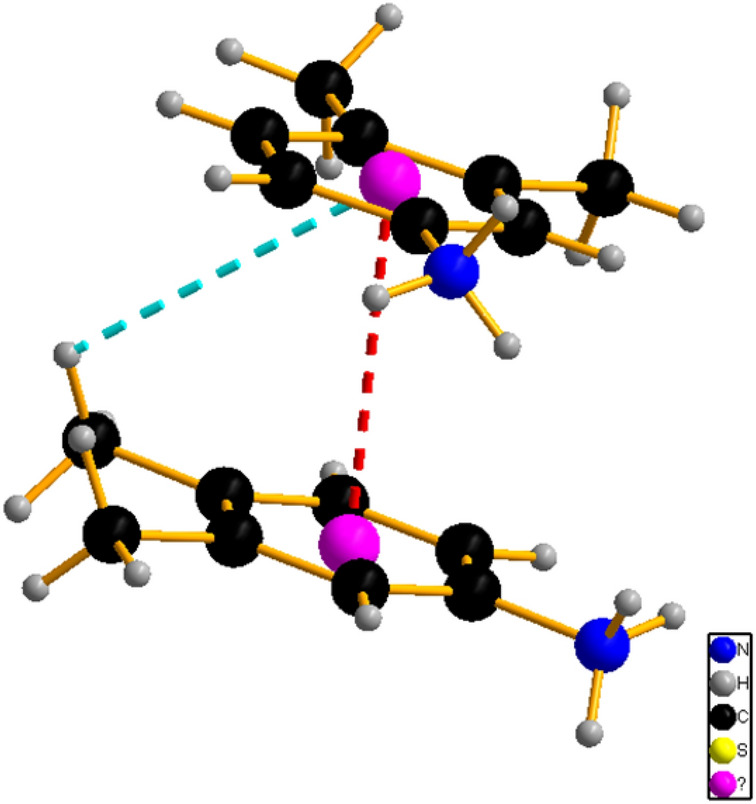
The CH–π interactions and the π-π stacking of {[Co(SCN)_4_] (C_8_H_12_N)_3_}·Cl.

The relative magnitude of various interatomic interactions, which can be stabilizing, determines the shape of coordination complexes (hydrogen bond, n-stacking). Because of the brief contacts between the phenyl ring atoms, the CH⋯π interactions have been found as a weak, attractive donor–acceptor type interaction between an acidic CH group and a basic π-system, which can impact molecule conformation and transition-state structure. Furthermore, the CH⋯π interactions (Fig. 5) between the CH frame and the aromatic rings with a distance of 3.876 Å improve the stability of the compound (1), adding more stability to the three-dimensional framework with a distance of 3.675 Å. There were no CH⋯π interactions or π–π stacking interactions observed in (**2**).

### Powder X-ray diffraction

**Figure 6:**
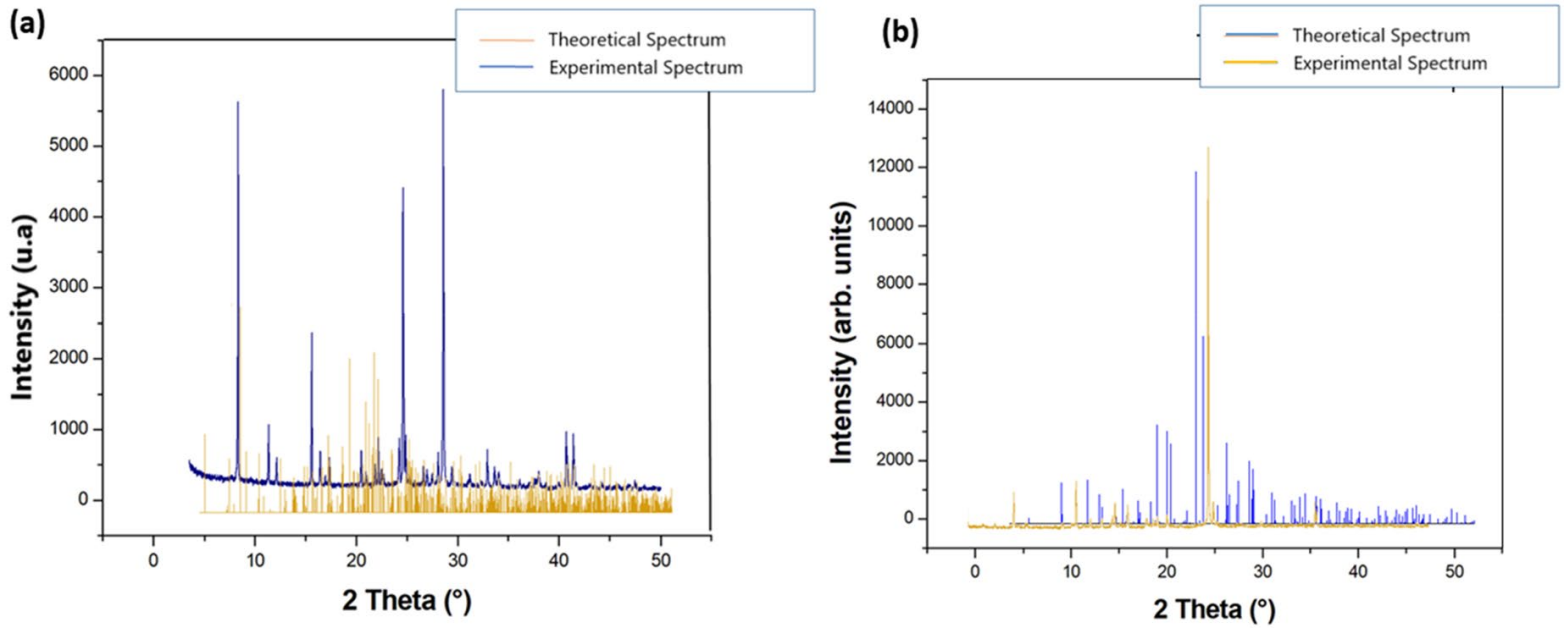
Final plot of the Rietveld refinement, showing the observed patterns of (**a**) {[Co(SCN)_4_] (C_8_H_12_N)_3_}·Cl and (**b**) {[Co(SCN)_4_] (C_5_ H_11_ N_3_)_3_}·2Cl.

The X-ray powder diffractograms for {[Co(SCN)_4_] (C_8_H_12_N)_3_}·Cl and {[Co(SCN)_4_] (C_5_ H_11_ N_3_)_2_}·2Cl are shown in Fig. 6. Some peaks with very low intensities, which could represent contaminants, cannot be indexed. The findings validated the identification of (**1**) and (**2**) as crystalline phases.

Figure 6 depicts the experimental and modeled PXRD patterns. As can be observed, the simulated X-ray powder diffraction pattern closely resembles the experimental pattern, with the majority of peak positions overlapping. We infer that the produced substance and the diffraction crystal data are both homogeneous.

### FT-IR spectrophotometry and assignments

**Figure 7:**
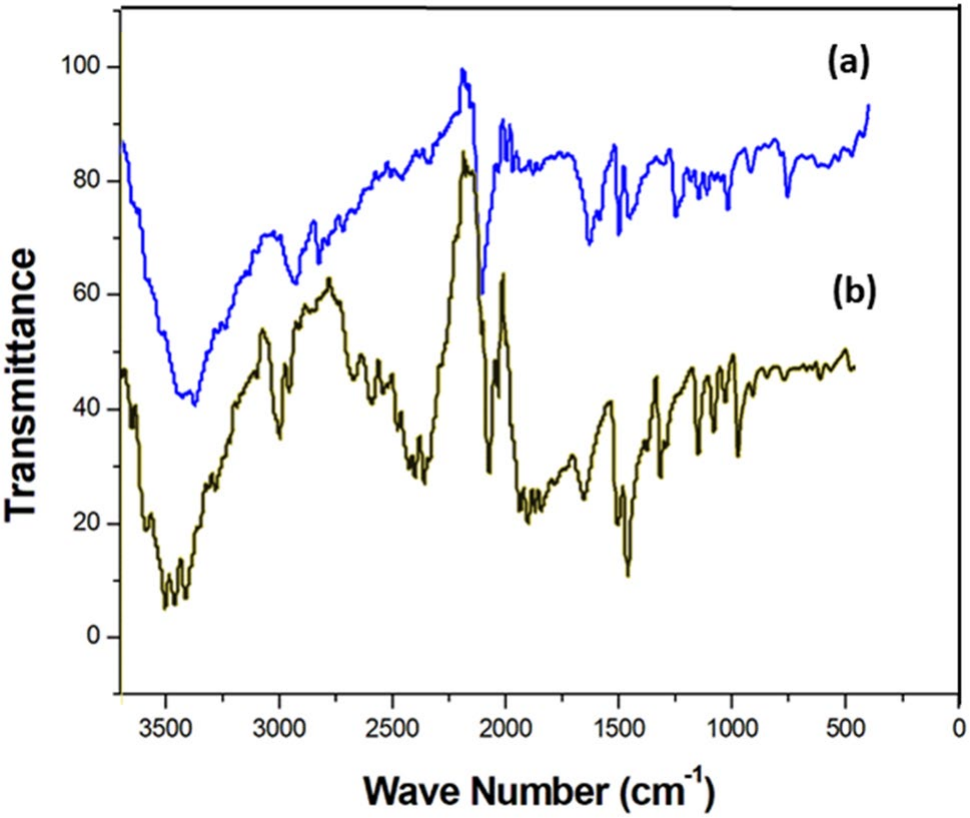
Infrared absorption spectra of (**a**) {[Co(SCN)_4_] (C_8_H_12_N)_3_}·Cl and (**b**) {[Co(SCN)_4_] (C_5_H_11_N_3_)_3_}·2Cl.

Figure 7 displays the IR spectrum of {[Co(SCN)_4_] (C_8_H_12_N)_3_}·Cl and {[Co(SCN)_4_] (C_5_H_11_N_3_)_2_}·2Cl.

The existence of the thiocyanate ligand and its binding mode to the Co(II) ion center for the creation of the anionic complex [Co(NCS)_4_] can be demonstrated by three distinctive vibrations.

The stretching vibration of the carbon–nitrogen triple bond of the thiocyanic ligand is responsible for the strong band at 2079 cm^−1^. The C-S bond stretching vibration is responsible for the weak band at 840 cm^−1^. The bending vibration of N–C–S is responsible for the weak band at 490 cm^−1^. Thiocyanate ligand binding to the Metal(II) center via its N-terminal atom is indicated by this vibration assignment. The identification of these bands to thiocyanate vibrations and the determination of their coordination mode is based on prior studies, such as those (C_2_N_6_H_12_) [Co (NCS)_4_]·H_2_O^64–67^.

The spectrum also exhibits cationic organisms' distinctive vibrations. The stretching vibrations of the organic and hydroxyl groups ν(N–H) and ν(C–H) correspond to the broad bands in the range 3600–2300 cm^−1^. Stretching vibrations ν(C=C) correspond to the band at 1504 cm^−1^. The CH_2_ deformation can be assigned to the band at 1450 cm^−1^.

The ring deformation is responsible for the bands at 1244 and 1180 cm^−1^. The CH_2_ twisting is responsible for the weak bands at 1166 and 1021 cm^−1^. The ring deformation is responsible for the weak band at 870 cm^−1^^6^.

### UV–Visible absorption spectral study

**Figure 8:**
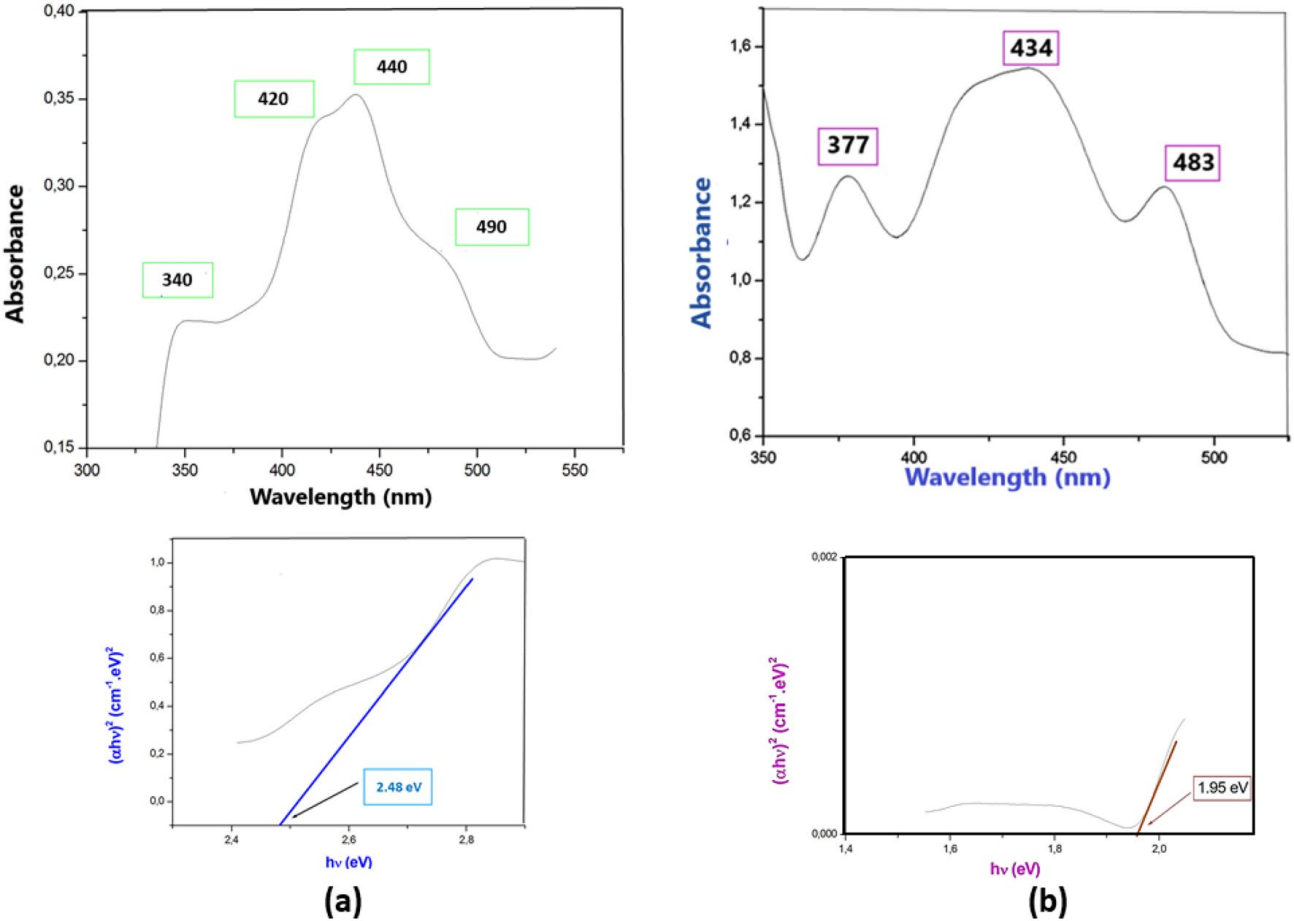
Solid state UV–Vis spectrum and TAUC representation of (**a**) {[Co(SCN)_4_] (C_8_H_12_N)_3_}·Cl and (**b**) {[Co(SCN)_4_] (C_5_ H_11_ N_3_)_3_}·2Cl.

At ambient temperature, the luminescence characteristics of {[Co(SCN)_4_] (C_8_H_12_N)_3_}·Cl and {[Co(SCN)_4_] (C_5_H_11_N_3_)_2_}·2Cl have been evaluated in the area [200–800 nm], as shown in Fig. 8. The compounds exhibit different luminescence behaviors; the four characteristic bonds for {[Co(SCN)_4_] (C_8_H_12_N)_3_}·Cl at 340, 420, 440, and 490 nm are assigned to charge transfer, n → π* π → π* d and → d*, transitions, respectively, and the three characteristic bonds for {[Co(SCN)_4_] (C_5_H_11_N_3_)_2_}·2Cl at 377, 434, and 483 nm are assigned to charge transfer.

The compound has a semiconductor characteristic, with Eg = 2.48 eV for (**1**) and 1.95 eV for (**2**), according to the Eg calculation (**2**). These characteristics are most likely owing to molecular interactions; charge transfer between core metals and their coordinated ligands; and in particular, the presence of thiocyanate anions, which may alter emission^68^.

### Thermoanalytical measurements

Measurements were made utilizing differential thermo-analysis and thermos-gravimetry (DTA-TG) concurrently to study the compounds' thermal characteristics.

In this regard, it was checked if a different, metastable modification of {[Co(SCN)_4_] (C_8_H_12_N)_3_}·Cl and {[Co(SCN)_4_] (C_5_H_11_N_3_)_2_}·2Cl can be obtained as recently reported for other ligands^69–71^.

**Figure 9:**
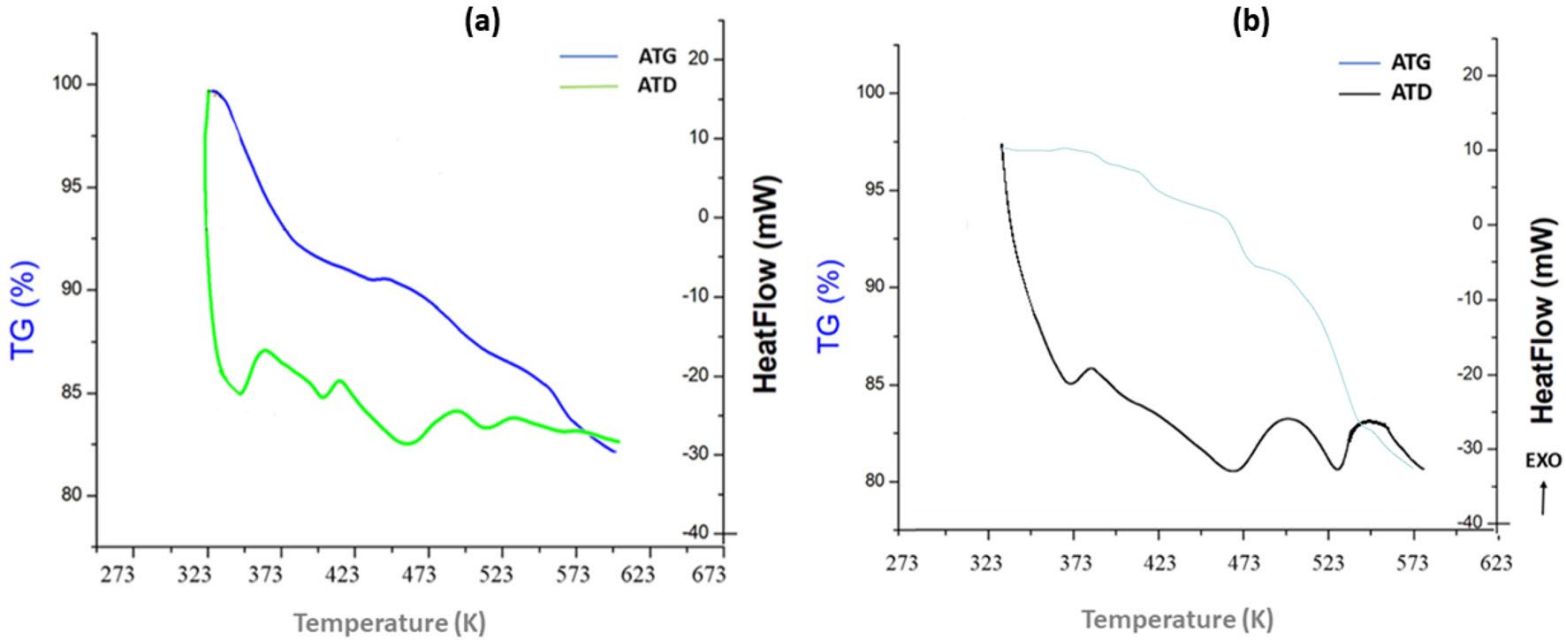
DTA/TG curves of (**a**) {[Co(SCN)_4_] (C_8_H_12_N)_3_}·Cl and (**b**) {[Co(SCN)_4_] (C_5_ H_11_ N_3_)_3_}·2Cl.

Figure 9a and b show the thermal curves of the two coordination compounds [(**1**) and (**2**), respectively]. With these structures, both compounds have similar thermal properties. It displays weight reduction between 347 and 480 K, complying with the decomposition of the organic part of the products due to the weakness of the N–H⋯Cl bonds as well as the departure of chloride molecules in the form of HCl. At higher temperatures, the DTA thermogram showed a series of successive endothermic peaks that correspond to the decomposition of the anionic part of the products, in the [490–547 K] range has the same variation for both compounds, it’s the decomposition of the resulting M(NCS)_4_ is carried out at a higher temperature^72^. The degradation is carried out in a wide temperature range. This can be explained by the strong Metal–Nitrogen coordination bonds.

### Antioxidant activity

#### DPPH scavenging activity

The synthesized compounds have been investigated using several tests to reveal the potential antioxidant behavior, three tests are carried out based on the reducing and chelating aspect regarding iron and scavenging activity of 2,2-diphényl-1picrylhydrazyl radical (DPPH·), Fenton model resume the reducing power of the compounds in presence of H_2_O_2_ to shift iron from (+ III) to (+ II) valence state, the chelating power was tested in presence of hydroxide and H_2_O_2_ to reduce Iron (+ II) to get iron (+ III) and hydroxyl radical the third test of the DPPH· is reduced to get DPPH-H which means the scavenging ability regarding the DPPH radical. These tests can judge if a compound has a direct or a secondary antioxidant activity when the crucial activity is detected respectively for DPPH· scavenging power and chelating or reducing the power of iron^73^.

**Figure 10:**
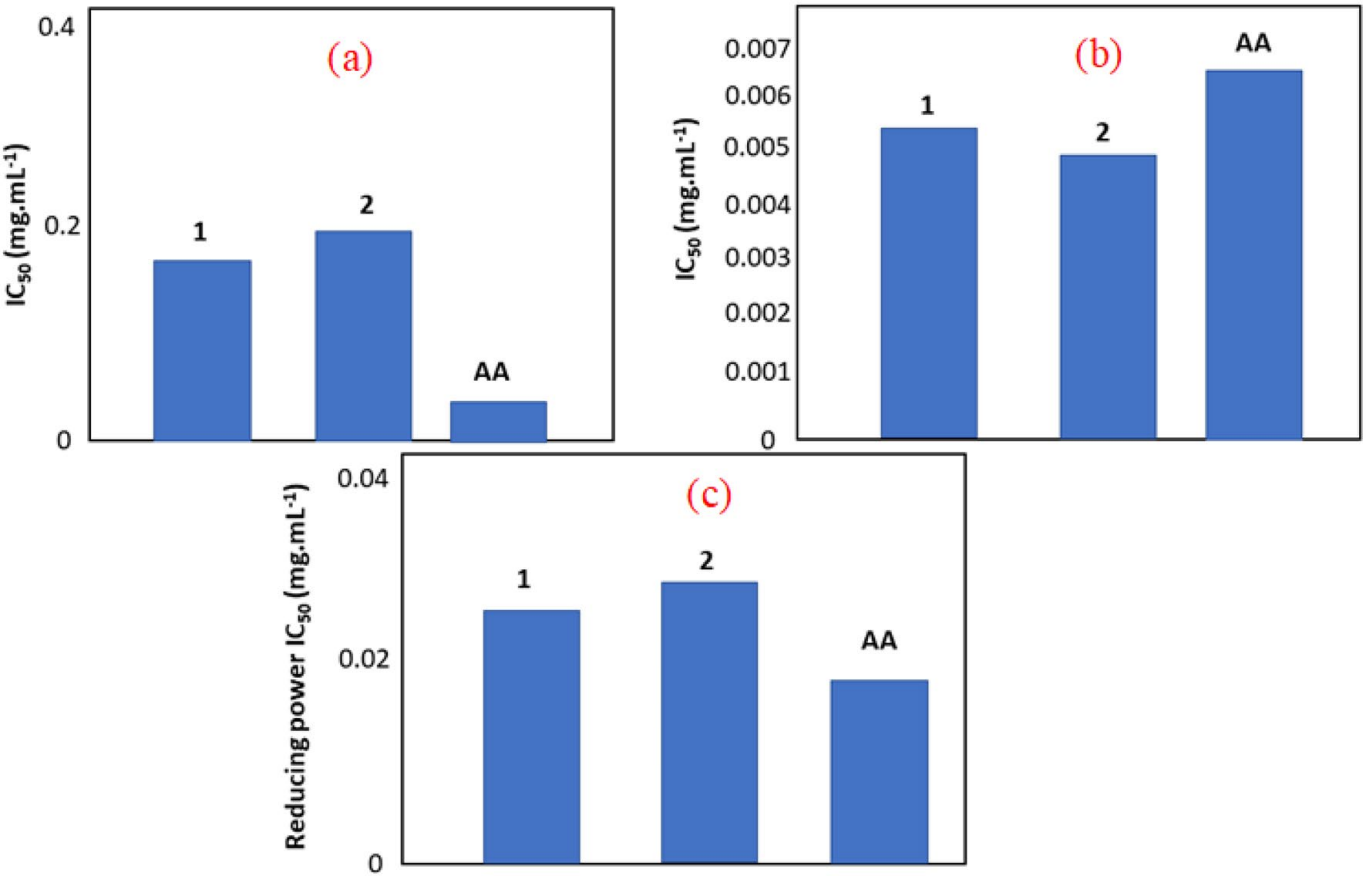
(**a**) Scavenging of DPPH· radical using the synthesized compounds (1: {[Co(SCN)_4_] (C_8_H_12_N)_3_}·Cl and 2: {[Co(SCN)_4_] (C_5_ H_11_ N_3_)_3_}·2Cl) compared to Ascorbic Acid (AA) as a standard. (**b**) Iron chelating power of the synthesized compounds (1: {[Co(SCN)_4_] (C_8_H_12_N)_3_}·Cl and 2: {[Co(SCN)_4_] (C_5_ H_11_ N_3_)_3_}·2Cl) compared to Ascorbic Acid (AA) as a standard. (**c**) Iron reducing the power of the synthesized compounds (1: {[Co(SCN)_4_] (C_8_H_12_N)}·Cl and 2: {[Co(SCN)_4_] (C_5_ H_11_ N_3_)_3_}·2Cl) compared to Ascorbic Acid (AA) as a standard.

The results are given in Fig. 10a show the high reducing capacity of the two synthesized organic–inorganic hybrid materials in the direction to cede a proton to get DPPH-H radical for scavenging the toxic radical DPPH· expressed as IC 50. The results show that the two tested nanomaterials have a good power relatively compared to ascorbic acid taken as a standard. Structurally the compounds have a graphing organic corona structure which gives a possibility to ensure the proton responsible to accomplish the reaction but the structure is not well flexible to the level to be at the same capacity of the standard used, the activity can be considered to be relative for the scavenging of the DPPH radical. As a comparable behavior detected regarding the scavenging activity of DPPH· radical for both compounds it may derive from the conserved part between them that host the protons which is represented by the amine groups.

#### Chelating power expressed to iron

Iron chelators are drugs that eliminate the excess iron from your body. The Food and Drug Administration (FDA) has approved two iron chelators for use in the United States. Deferoxamine (Desferal®) is normally given as a subcutaneous (under the skin) infusion through a small portable pump approximately the size of a CD player.

The capacity related to the chelating achievement of the reaction was presented in Fig. 10b, the histograms show that both synthesized compounds work as an excellent agent better than the standard AA expressed by IC 50^74^ the values are almost at the 5 folds more active than the standard, this power theoretically related to the flexibility of the tested compounds to collect the released electrons from the reaction. It is important to mention that the compound **1** and **2** have, literary, the same capacity in terms of value which can be explained by a commune inorganic part of structure conserved between them, the examination of the structure let to conclude about the presence of the inorganic part of the compounds based of Co center add to the (SCN)_4_ to be responsible for this power for collection of electrons.

#### Reducing the power of iron

The test is based on the capacity to cede electron to run the reciprocity reaction of the previous test, it can be written like the following: Fe^3+^ + e^–^ = Fe^2+^. At this level Fig. 10c shows a synergy between compound 1 and the standard having a comparable activity, the compound 2 present also has a good capacity to cede the electron. These results reveal that the transformation of Fe^3+^/Fe^2+^ ability, in presence of compound **1** and **2**, have been enough to mark the iron reduction.

The equation involves the capacity of the inorganic part to give the requested electron to ensure the reaction. The dissociation of the compounds releases the chloride in the solution with is the only difference.

In the conclusion, it is clear to mention that compounds **1** and **2** can be a powerful secondary antioxidant agents working with the capacity to release and give electrons at the demand of the biological cell.

Both compounds having a relative DPPH radical scavenging activity coupled with chelating iron ability seems to be a primary antioxidant agent^75^. The two compounds have a little bit of difference regarding the performed antioxidant tests with different modalities can be considered to be with a potential pharmacological application in this direction.

#### Hirshfeld surface study (HSA)

**Figure 11:**
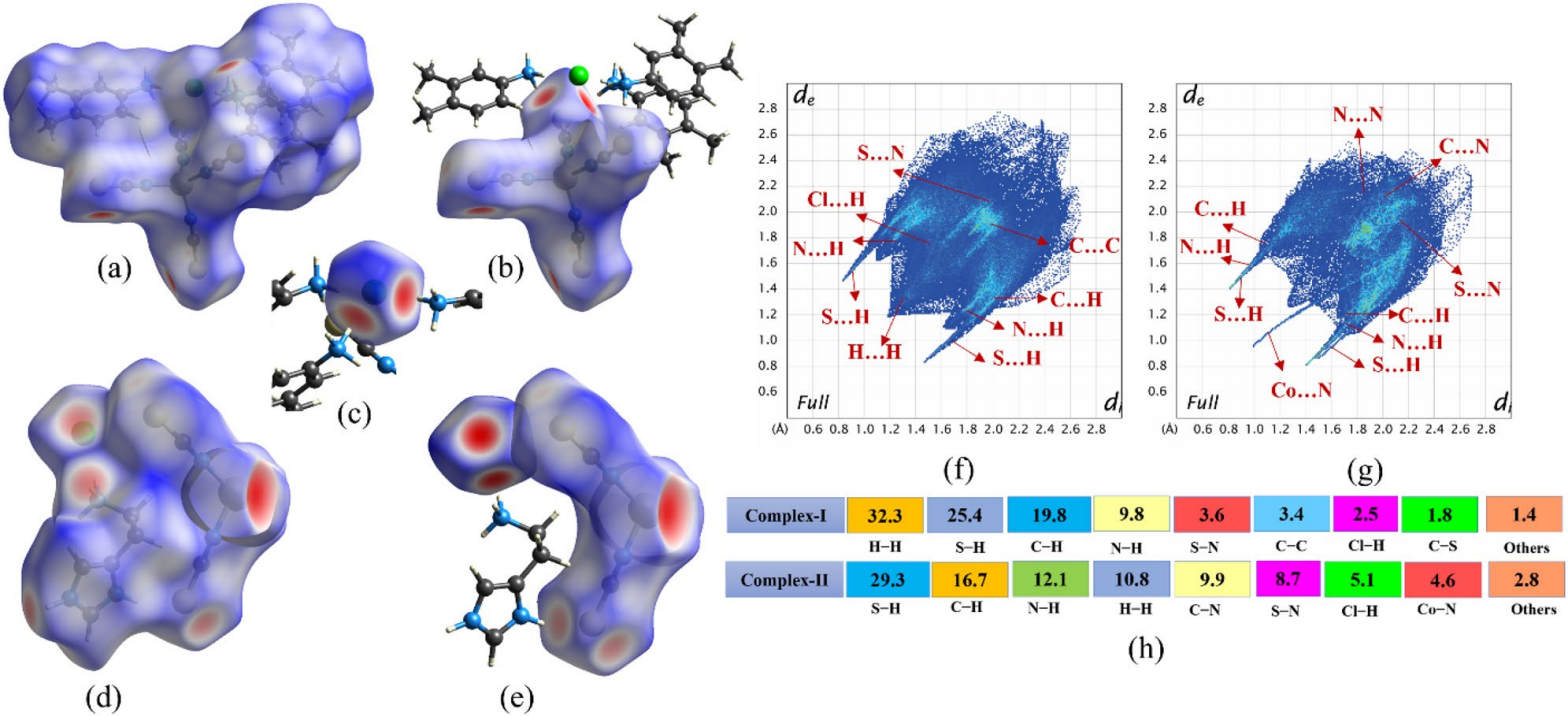
The dnorm map of Hirshfeld surface analysis and their Fingerprint plots with contribution (%) for both (**a-c,f**) complex-I and (**d-e,g**) complex-II.

**Figure 12:**
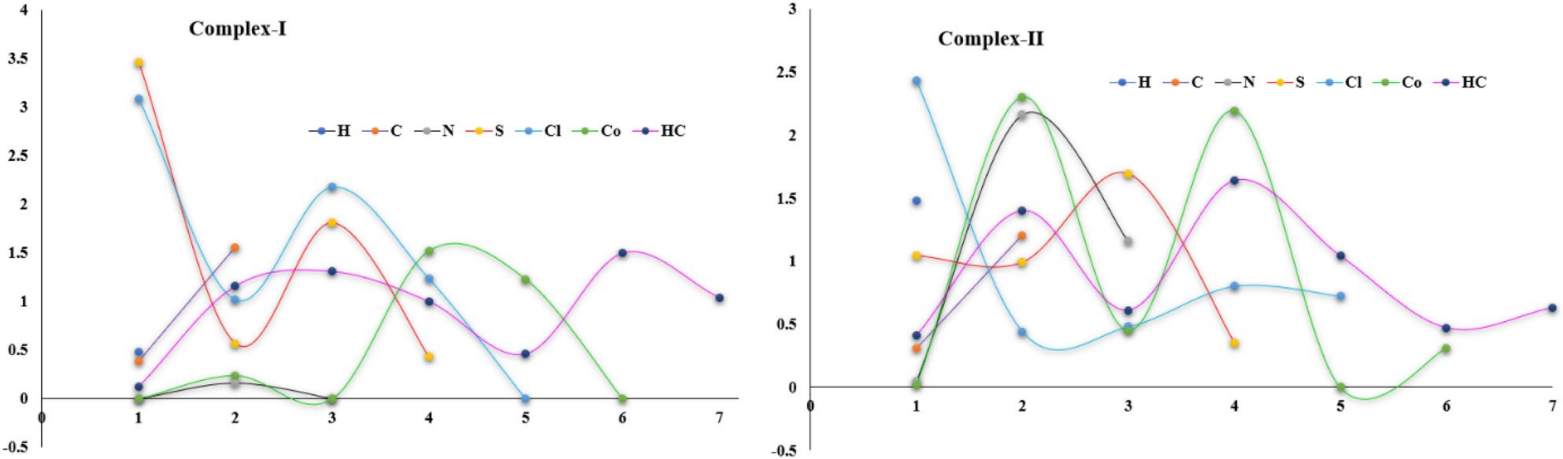
The correlation map shows the enrichment ratio of both complexes.

With the help of the HSA study, the stabilizing interactions in the crystal packing such as donor–acceptor groups, hydrogen bonds, and π⋯π interactions can be identified as well as visualized. The d_norm_ map of both Co-metal complexes is shown in Fig. 11; in which, the dark red spots appear over the sulfur, nitrogen, and chlorine atoms in both metal complexes due to that the atoms are the nearest external nuclei from the acceptor. These d_norm_ maps are confirmed that N, S, and Cl atoms are forming the N‒H⋯S and N‒H⋯Cl type of intermolecular interactions in both metal complexes. Also, the red spot over the Cl atom indicates their bonding engagement with three Amine groups. Further, the red surface over the Co-metal in the complex-**II** confirms the symmetry-related bonding and Co⋯S interaction which is not present in the complex-**I**. Not only hydrogen bonding but also visualize weak bonding interactions in the presence of the blue and white surface over the d_norm_ map of Hirshfeld surfaces. Secondly, the quantitative view of non-covalent interactions in the crystal packing can be shown in the 2D fingerprint plots with the percentage of contribution. Here, more than 25% of the interactions are S⋯H contributions in both complexes. In comparing both complexes, the contribution of H⋯H and C⋯H interaction in the complex **I** is much higher than the complex **II**; whereas N⋯H and N⋯S contribution of **I** is lower than the **II**. On comparing the Fingerprint plots of both molecules, the sharp spikes fully appear on S⋯H contacts and small spikes are also found on N⋯H contacts; notably, the Co⋯N contacts especially appear as a single spike in the complex-II which is not in the complex-I. These sharp spikes indicated that the dominant interactions are cyclic H-bond character of the sulfur and nitrogen groups in the metal complexes. Further, the strong cam-lobe shape exhibited for H⋯H interactions in the I which is weak in the II and all other interactions are under to wing shape in the fingerprint plots. On the other hand, the enrichment ratio (E_AB_) is also derived from the HS study (Fig. 12). The value of enrichment ratio for the S⋯H and Cl⋯H are found to be greater than the other pairs (hydrogen pairs) as well as Co⋯S, S⋯N, and S⋯Cl pairs also higher than other non-hydrogen pairs in both complexes which indicates that these pairs exhibit a larger tendency to form interconnects in the solid-state phase.

#### Molecular electrostatic potential map

**Figure 13:**
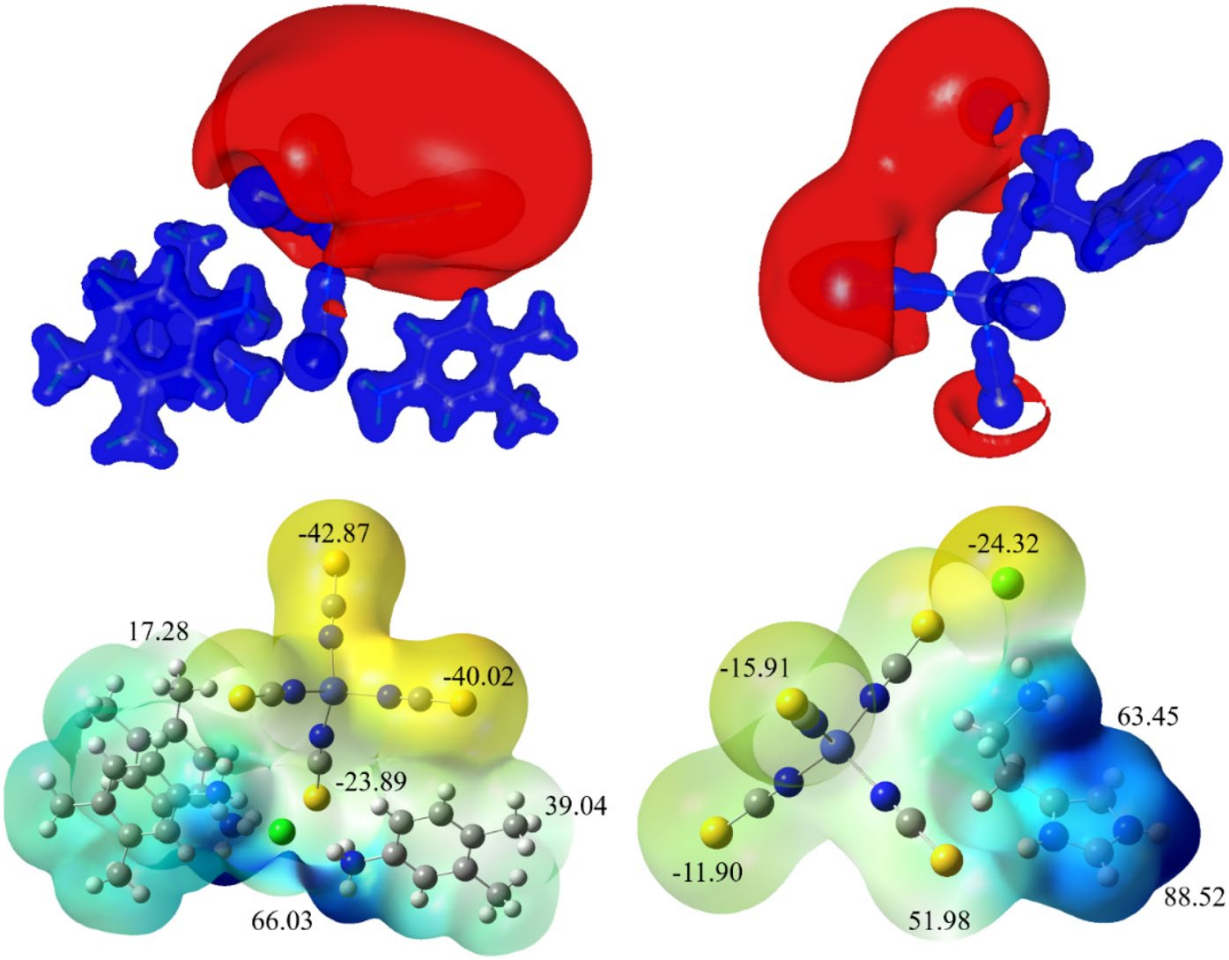
Electrostatic potential map of both metal–organic complexes.

In general, the molecular electrostatic potential map helps to understand the electron density distribution over the surface of a molecule. It also allows for revealing the molecular reactivity, electrophilic and nucleophilic sites^76^. Here, the computed MESP map for both metal complexes is shown in Fig. 13; In which, the high vicinity of electronegative surface is noticed on the sulfur and chlorine atoms in both complexes. The color range starts from most negative to most positive (red–orange–yellow–blue). The indicates electron lacking, and yellow/red is an electron-rich region. Therefore, the two different maps of electrostatic potential and total electron density reveal the most concentrated region for the electrophilic attack, likely, non-covalent interactions during the crystal packing. Further, the ESP minima and maxima were also calculated and the high negative ESP value (in kcal/mol) was found around sulfur and chlorine atoms whereas the high negative ESP is near methyl groups. From this, we can highlight the non-covalent interaction region for both molecules.

#### HOMO and LUMO

**Figure 14:**
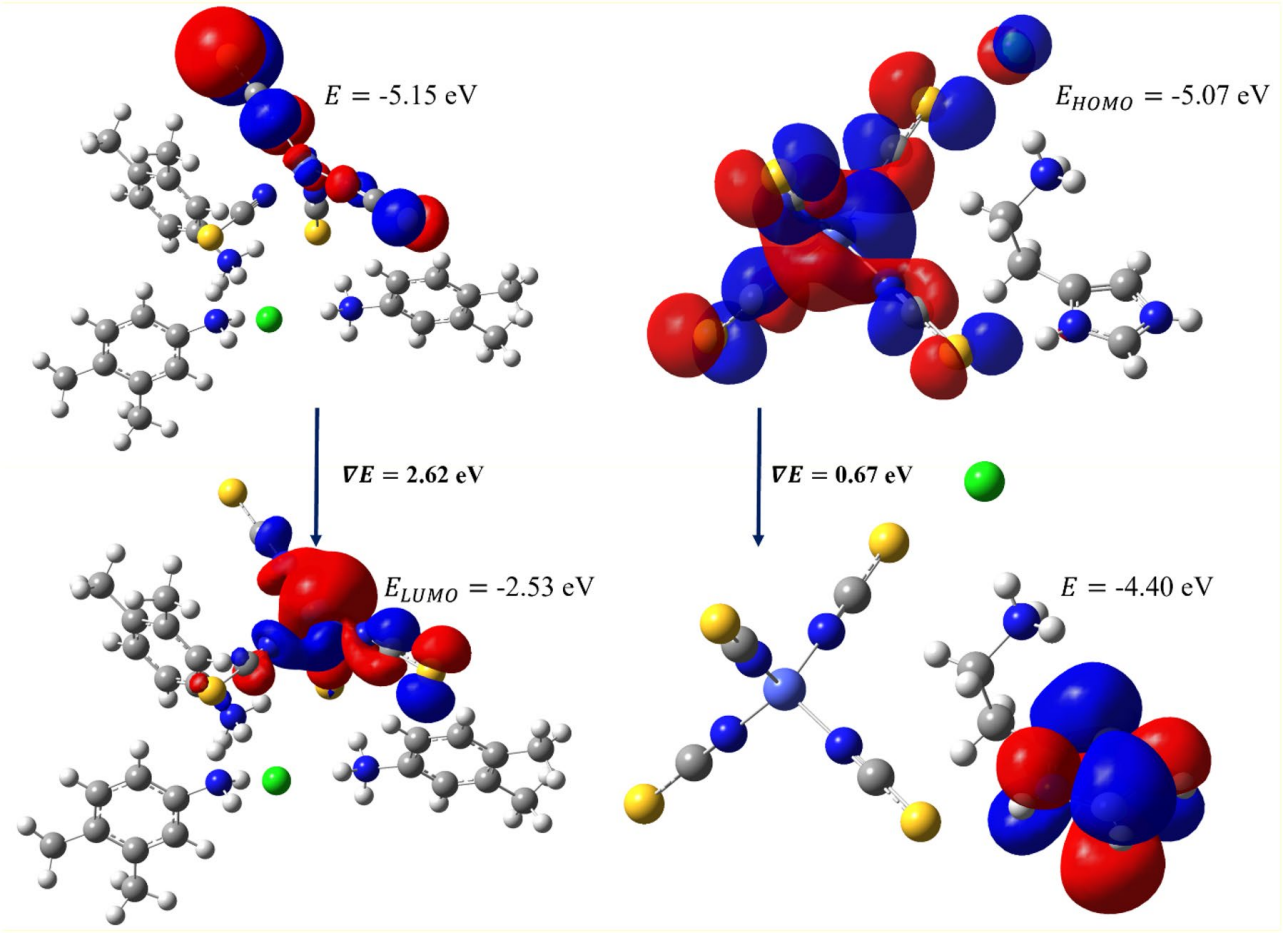
The HOMO and LUMO map of both metal complexes, the map was drawn at 0.02.

The highest occupied molecular orbital (HOMO) and lowest unoccupied molecular orbital (LUMO) are used to calculate the electronic characteristics of molecular structures in the frontier molecular orbital analysis^47–52^. In the present investigation, all the above-desired chemical properties and global reactivity descriptors are calculated and their values are given in Table 2. The band gap of both complexes exhibits a small value which confirms the chemical activity, optical polarizability, and low kinetic stability and also shows that the compound can favor the biological activity of the compound. In comparison with both molecules, complex **II** is much lower than **I**. Moreover, the HOMO and LUMO energy allow for to calculation of the reactivity descriptors such as ionization potential and electron affinity. This ionization potential and electron affinity are used to calculate the global hardness, electronegativity, and electrophilicity. The iso-surface HOMO and LUMO maps of both complexes are shown in Fig. 14; in which, the localization and delocalization of orbitals help to understand the electronic transitions in the coordination complexes. The HOMO and LUMO are mostly localized as well as delocalized on the metal and sulfur regions in both complexes. Notably, the HOMO is also localized on the chlorine atom in complex-II whereas it does not appear in the I. The high delocalized LUMO is observed around the metal and sulfur regions this may be the strong metallic coordination bonding. Both metal complexes are equivalent based on this molecular orbital study, and they may act as intra-ligand charge transfer metal complexes.

**Table 2:** Calculated global reactivity properties of the molecule.

Global reactivity descriptors	DFT energy (eV)	
	Complex-**I**	Complex-**II**
Band gap	2.62	0.67
HOMO energy	− 5.15	− 5.07
LUMO ENERGY	− 2.53	− 4.40
Ionization potential I = − E_HOMO_	5.15	5.07
Electron affinity A = − E_LUMO_	2.53	4.40
Global hardness η = (I − A)/2	1.31	0.33
Electronegativity χ = (I + A)/2	3.84	4.74
Electrophilicity ω = μ^2^/2η, μ = − χ	5.62	33.62

#### NLO and NBO analysis

Nowadays, the role of non-linear optical activity of the organic and metal–organic complexes is one of the significant phenomena in the applications of signal processing, optical communications, optical switching, ultrafast response, laser projection display technologies, and data storage^77^. Moreover, according to the studies performed early that shows metal-based organometallic complexes carry outstanding electrochemical and optical properties than the organic and inorganic compounds^78^. Therefore, the NLO properties of both Co-metal organic complexes have been reformed by quantum chemical calculation at the B3LYP/LANL2DZ/6-311G** level of basis sets in the gaussian 09 software package. In which, the total dipole moment (µ), the average and anisotropy of polarizability (α & ∆α), and hyperpolarizabilities (β) were calculated to characterize the NLO properties of the selected molecules (Table 3). The calculated values are converted from the atomic units (a.u) to electrostatic units (esu) using the conversion factor (α: 0.1482 × 10^–24^ esu & β: 8.6393 × 10^–33^ esu). Urea was the first organic compound which was studied the NLO properties and now it is used as the standard reference for new NLO materials^79^. Not only urea, some of the articles compared KDP as a reference^80^. The dipole moment of both metal–organic complexes is 25.30 and 11.69 Debye respectively; here, the complex-I exhibits stronger interactions than I confirmed from the structural analysis. Importantly, the computed dipole moment of Co-metal complexes is higher than urea as well as KDP. The hyperpolarizability of these complexes is 11.029 (I) and 4.580 × 10^–30^ respectively; it shows ~ 55 and ~ 22 times higher than the urea and ~ 14 and ~ 6 times higher than the KDP. Also, these values are much higher than previously reported similar kinds of cadmium and zinc complexes^81,82^. Therefore, these metal complexes are more potent as well as effective NLO materials. As a result of the large β-value, organometallic compounds are an appealing item for future nonlinear optical property studies and useful material for NLO properties.

**Table 3:** Dipole moment (µ) in Debye (D), polarizability (α), and hyperpolarizability (β) of the titled compounds using the base level of DFT/LANL2DZ (for Co-metal)/B3LYP 6-311G** (for all atoms except Co) method.

	Complex-I	Complex-II	Urea/KDP
µx	− 20.31	0	
µy	− 29.42	0	
µz	− 4.48	32.62	
µ_total_	36.03	32.62	1.3197/6.03
α_XX_	− 3.393 × 10^–23^	1.045 × 10^–23^	
α_XY_	− 7.223 × 10^–24^	1.224 × 10^–24^	
α_YY_	− 4.556 × 10^–23^	1.291 × 10^–23^	
α_XZ_	− 1.089 × 10^–24^	5.829 × 10^–25^	
α_ZZ_	− 4.505 × 10^–23^	1.002 × 10^–23^	
α_YZ_	1.791 × 10^–24^	1.318 × 10^–24^	
α_Total_	− **4.152 × 10**^**–23**^	**1.113 × 10**^**–23**^	
∆α	1.138 × 10^–23^	2.707 × 10^–23^	
β_XXX_	− 3.642 × 10^–30^	− 2.085 × 10^–31^	
β_XXY_	− 3.090 × 10^–30^	− 5.007 × 10^–31^	
β_XYY_	− 9.850 × 10^–31^	− 9.979 × 10^–31^	
β_YYY_	− 5.226 × 10^–31^	− 2.041 × 10^–30^	
β_XXZ_	− 1.211 × 10^–30^	− 4.270 × 10^–32^	
β_XYZ_	2.620 × 10^–31^	− 2.132 × 10^–31^	
β_YYZ_	− 6.004 × 10^–32^	− 2.056 × 10^–31^	
β_XZZ_	− 8.085 × 10^–31^	2.392 × 10^–32^	
β_YZZ_	− 1.206 × 10^–31^	7.181 × 10^–31^	
β_ZZZ_	8.576 × 10^–32^	4.280 × 10^–30^	
β_total_	**11.029 × 10**^**–30**^	**4.580 × 10**^**–30**^	**0.1947/0.732 × 10**^**–30**^

Significant values are in bold.

The effect of covalency and hybridization in a molecular system can be well examined by natural bond orbital (NBO) and it also helps to study the intermolecular orbital interactions such as hydrogen bond and strong van der Waals interactions^83^.

The coordination bonds and their stability in the organometallic compounds are characterized by the strength of interactions in the synthesized complexes^81,82^. Therefore, NBO analysis of both metal complexes was performed at same level as the DFT method. The most important interactions between donor and acceptor orbitals obtained in the metal–organic complexes are shown in Table 4, which is formed by overlapping the orbitals of π → π*, σ → σ*, and σ → π*, resulting intermolecular charge transfer to stabilize the molecular system. The n → π* interactions are found to be the greatest stabilization energy among all other interactions in the corresponding system. In both complexes, there are two interactions are seemed to be stronger, the stabilization energies are shown in Table 4. In which, the N‒H⋯Cl and N‒H⋯S interactions in the complex II carried high stabilization energy than the same interaction in the complex-I. However, all the tabulated hyper conjugative interactions are found to be higher which leads to elongating their corresponding bonds.

**Table 4:** The NBO analysis of metal–organic complexes.

Type of n_A_	Electron configuration of n_A_	Type of orbital interaction	E^(2)^ (kcal/mol)	Occupancy of σ_H-D_*	Bond order of σ_H-D_^b^
**Complex-II**					
LP(3)S_2_	s (0.35%) p 99.99 (99.65%)	BD*(1) N_1B_‒H_1I_	8.96	0.74	0.07
LP(3)Cl_1_	s (12.29%) p 7.14 (87.71%)	BD*(1) N_1B_‒H_1G_	14.23	0.61	0.12
**Complex-I**					
LP(4)Cl_1_	s (11.99%) p 7.34 (88.01%)	BD*(1) N_3_‒H_3C_	53.1	0.54	0.11
BD*(1)S_1_‒C_6_	s (14.62%) p 5.84 (85.38%)	BD*(1) N_1_‒H_1N_	6.87	0.02	0.03
BD*(1)S_1_	s (0.25%)p 99.99 (99.75%)	BD*(1) N_1_‒H_1N_	11.4	0.08	0.06

#### Quantum theory of atoms in molecules

**Figure 15:**
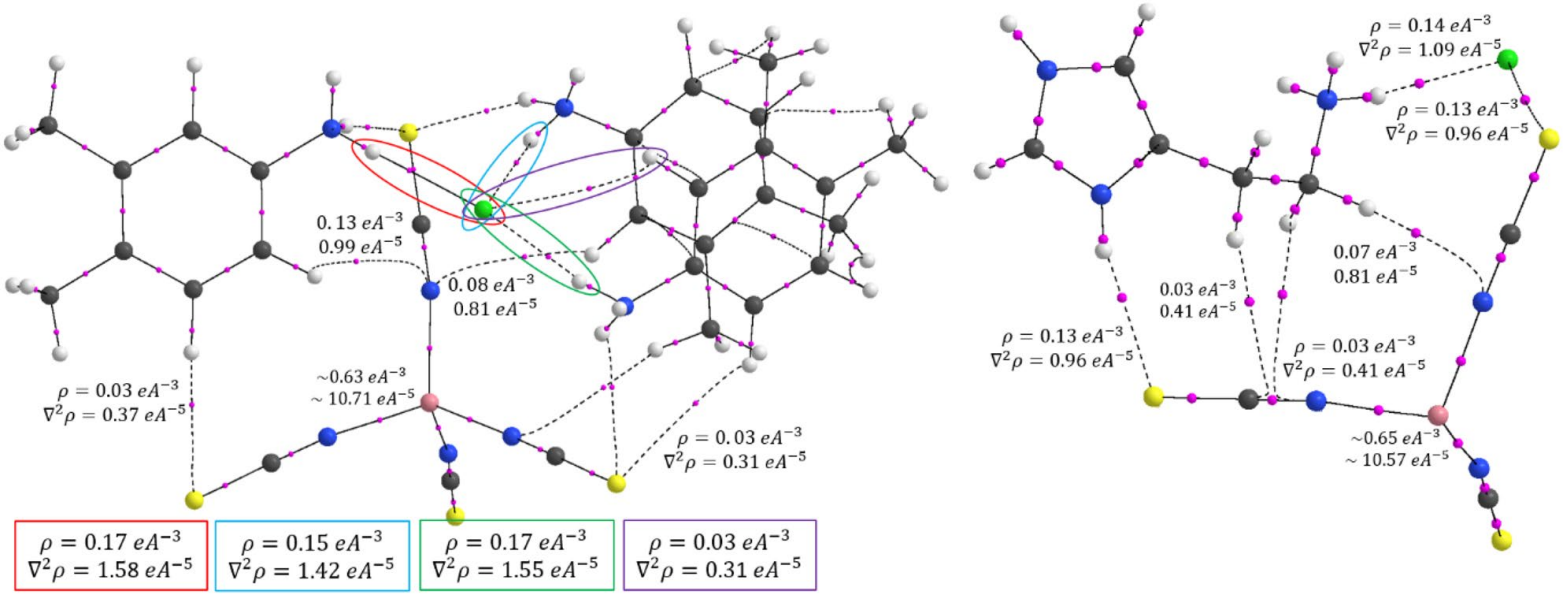
The bond critical point (bcp) map with their topological properties.

**Figure 16:**
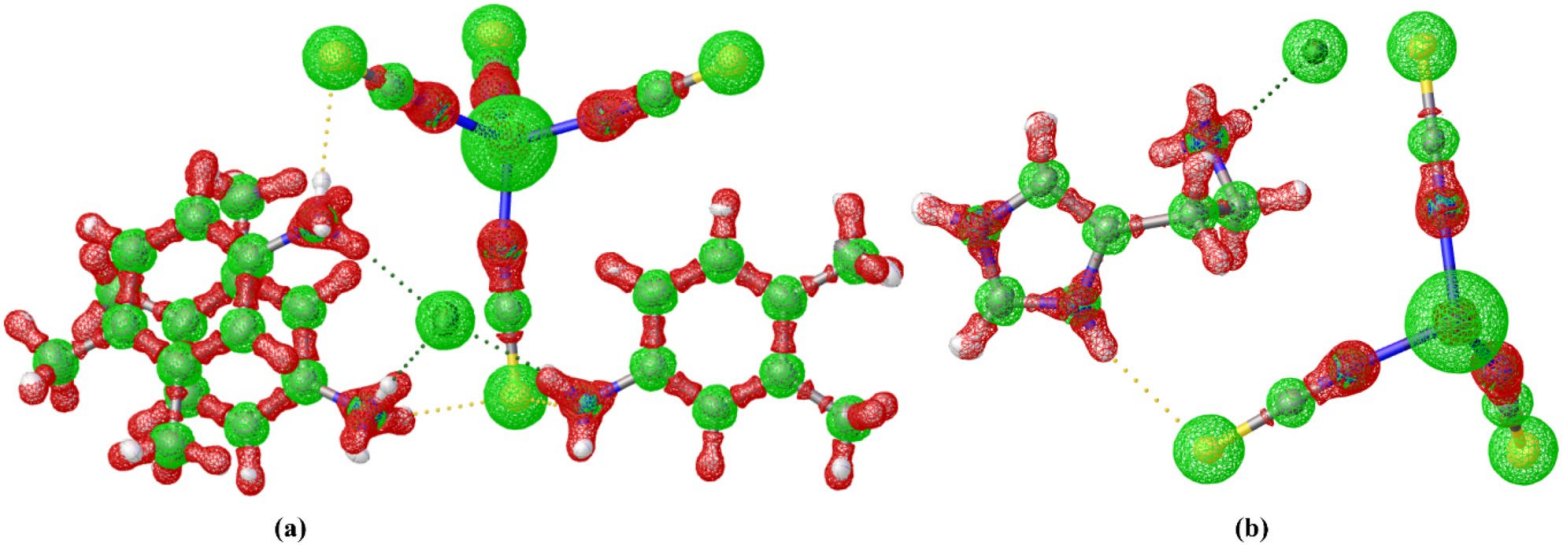
Laplacian of electron density map of both complexes (**a**, **b**) map was drawn at 0.1 Å.

Nowadays, the Bader theory of atoms in molecules has been widely used to determine the different types of chemical bonding like covalent bonding, and non-covalent interactions in various molecular systems^84^ like organic compounds^85^, organic salts/cocrystals^86^, metal–organic complexes^87^ and protein–ligand complexes^88^. These interactions are calculated from the topological properties such as electron density, and Laplacian of electron density at the bond critical points (BCPs). In QTAIM, the presence of a chemical bond between a pair of atoms is determined by the appearance of a critical point with rank; in which, the (3, −1) type of bond critical point search was executed. Figure 15 shows the bond critical point with their electron density and Laplacian electron density; in which, the N‒H⋯Cl, N‒H⋯S, C‒H⋯N and C‒H⋯C type of interactions are shown in dotted lines. Here, the small pink color sphere indicates the bond critical point of the corresponding chemical bond. The electron density ρ_cp_(r) and Laplacian of electron density ∇^2^ρ_cp_(r) of N‒H⋯Cl and N‒H⋯S interactions show high electron density and positive Laplacian of electron density values than other interactions in the molecule. The closed-shell interactions were verified by the lower amount of electron density and positive Laplacian of electron densities. Importantly, in the Co-metal coordinates with four nitrogen atoms, the ρ_cp_(r) and ∇^2^ρ_cp_(r) values of Co‒N bond is 0.63/0.65 eÅ^−3^ and 10.??71/10.??57 eÅ^−5^ respectively, these values are smaller than the previously reported Co‒N bond due to different bonding orientation and basis set effect^89^. Also, the topological properties of intermolecular interactions were highly correlated with the geometry of interactions. The 3D Laplacian of electron density map was drawn (Fig. 16) with the help of a wave function file generated using the NoSpherA2 module^90^ and ORCA 4.2.1^91^ in the Olex 1.5 software^92^.

**Figure 17:**
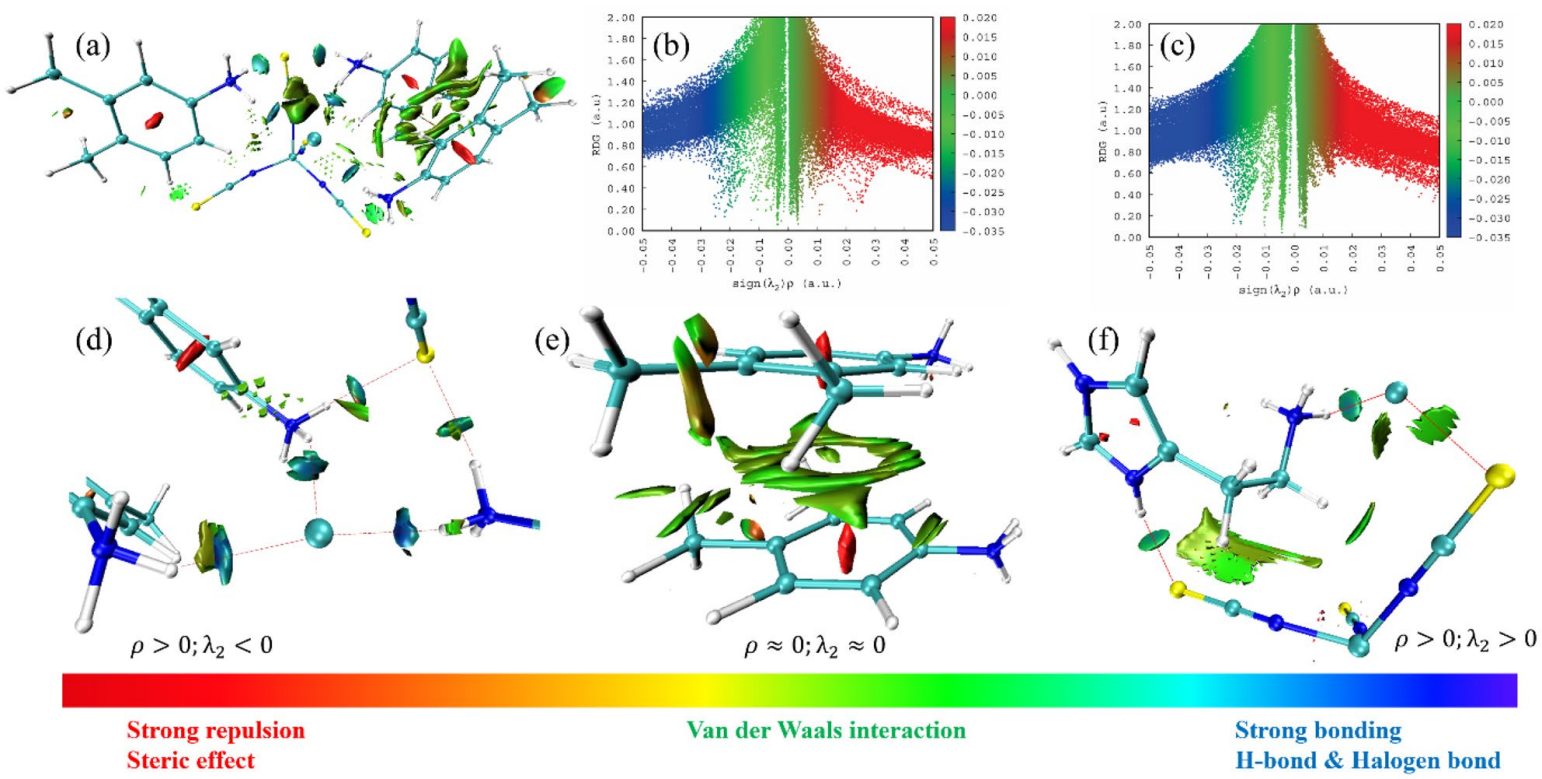
Non-covalent interaction isosurface map and their RDG plot.

Johnson and his coworkers developed a method^93^ called non-covalent interaction analysis which allows for characterizing the intermolecular interactions and hydrogen bonding as well, it helps to analyze the weak interactions in the molecular system. The reduced density gradient (RDG) is a scalar quantity, giving the strength of intermolecular interactions. The RDG was mapped against the electron density sign (λ_2_)ρ; here, the repulsive interactions are confirmed from the sign (λ_2_)ρ value greater than zero whereas the attractive interactions are from lesser than zero. The colored RDG map shows the strong interactions are in blue, weak van der Waals interactions are in the green color and strong repulsion forces are in red. Figure 17a–f shows the NCI plot and isosurface map of non-covalent interactions in both metal complexes. In which, the blue color surface in between two interacting atoms reveals strong bonding, the blue-green color surface indicates van der Waals and stacking interactions (X‒H⋯π) and the red surface within the center of the rings exhibits strong repulsion forces, resulting from steric effect and these all interactions are confirmed from the NCI-RDG scatter plots.

## Conclusion

The preparation and investigation of novel coordination compounds, {[Co(SCN)_4_](C_8_H_12_N)_3_}·Cl and {[Co(SCN)_4_] (C_5_H_11_N_3_)_2_}·2Cl, has been described and characterized. For both complexes, the metal center was found to be tetracoordinate with four NCS entities to establish a tetrahedral geometry. The intermolecular cohesion is ensured by N–H⋯S hydrogen bonds and π⋯π stacking interactions. Add to the optical properties were investigated by FT-IR absorption measurement and solid-state Ultra-violet measurements which gives an important optical behavior. A thermal study by differential thermal analysis (DTA) and thermogravimetric (GTA) highlights the ability and the decomposition ranges. Additionally, the antioxidant assay also proves a high efficiency compared with ascorbic acid.

The intermolecular interactions and crystal packing were analyzed with the help of different types of mapping that explains their contributions in the solid-state using HS study. The fingerprint plot allows us to characterize the predominant interactions that are dominated by N‒H⋯S and N‒H⋯Cl type of interactions. The electron donor and acceptor regions in the Co-metal organic complexes were identified using electrostatic potential maps. The NLO analysis predicts that the Co-metal complexes can be used as NLO material in different applications. The molecular orbital analysis and global reactive descriptors confirmed their chemical reactivity. The noncovalent interaction and QTAIM analysis help to uncover the nature of interactions in the crystal phase.

## Supplementary Information

Supplementary Tables.

## Data Availability

Data generated or analyzed during this study are included in this published article [and its supplementary information files]. A CCDC Deposition Number 2061205 and 2131386 contain the supplementary crystallographic data for (**1**) and (**2**). This data can be obtained free of charge via http://www.ccdc.cam.ac.uk/conts/retrieving.html, or from the Cambridge Crystallographic Data Center, 12 Union Road, Cambridge CB2 1EZ, UK; fax: (+ 44) 1223-336-033; or email: deposit@ccdc.cam.ac.uk.
