# Supplementary material for: Self-assembly of new cobalt complexes based on [Co (SCN)4], synthesis, empirical, antioxidant activity, and quantum theory investigations
Source: Sci Rep. 2022 Sep 22;12:15828. doi: 10.1038/s41598-022-18471-7 (PMC9500081; doi:10.1038/s41598-022-18471-7)
Supplement: Supplementary file 1 — Supplementary Tables. [file 41598_2022_18471_MOESM1_ESM.docx]

**Supplementary material**

**Table S1**: Selected bond lengths and bond angles of the two cobalt complexes in the title compound.

| [Co(SCN)_4_] (C_8_H_12_N)_3_ Cl | | | |
| --- | --- | --- | --- |
| **Atoms** | **Distance** | **Atoms** | **Angles** |
| Co1—N4 | 1.955 (4) | N4—Co1—N3 | 105.93 (16) |
| Co1—N3 | 1.958 (4) | N4—Co1—N2 | 115.94 (15) |
| Co1—N2 | 1.959 (4) | N3—Co1—N2 | 107.62 (15) |
| Co1—N5 | 1.962 (4) | N4—Co1—N5 | 105.14 (15) |
| S1—C9 | 1.631 (5) | N3—Co1—N5 | 113.93 (15) |
| S3—C11 | 1.635 (5) | N2—Co1—N5 | 108.48 (16) |
| S2—C10 | 1.637 (5) | C9—N2—Co1 | 170.8 (4) |
| S4—C12 | 1.633 (5) | C10—N3—Co1 | 173.8 (4) |
| [Co(SCN)_4_] (C_5_ H_11_ N_3_)_3_, 2Cl | | | |
| Co1—N5^i^ | 1.949 (3) | N5^i^—Co1—N5 | 115.60 (19) |
| Co1—N5 | 1.949 (3) | N5^i^—Co1—N6 | 113.29 (11) |
| Co1—N6 | 1.962 (5) | N5—Co1—N6 | 113.29 (11) |
| Co1—N4 | 1.986 (4) | N5^i^—Co1—N4 | 106.20 (11) |
| S1—C6 | 1.648 (5) | N5—Co1—N4 | 106.20 (11) |
| S2—C7 | 1.635 (4) | N6—Co1—N4 | 100.54 (18) |
| S3—C8 | 1.624 (6) | C6—N6—Co1 | 167.5 (4) |
| N4—C6 | 1.161 (6) |  |  |
| Symmetry code : (i) *x*, −*y*+1/2, *z*. | | | |

**Table S2** : : [Hydrogen-bond geometry (Å, °)](about:blank)

| [Co(SCN)_4_] (C_8_H_12_N)_3_Cl | | | | |
| --- | --- | --- | --- | --- |
| D—H···A | D—H | H···A | D···A | D—H···A |
| N1—H1A···S3^i^ | 0.90 (2) | 2.42 (2) | 3.317 (4) | 174 (4) |
| N1—H1B···S1 | 0.90 (2) | 2.46 (2) | 3.342 (4) | 165 (4) |
| N1—H1C···Cl1 | 0.90 (2) | 2.24 (2) | 3.127 (4) | 169 (4) |
| N1A—H1D···S1 | 0.92 (2) | 2.37 (2) | 3.259 (4) | 162 (3) |
| N1A—H1E···Cl1 | 0.91 (2) | 2.25 (2) | 3.158 (4) | 178 (4) |
| N1A—H1F···S4^ii^ | 0.90 (2) | 2.40 (2) | 3.282 (4) | 166 (4) |
| N1B—H1G···Cl1 | 0.92 (2) | 2.18 (2) | 3.076 (4) | 166 (4) |
| N1B—H1H···S4^i^ | 0.91 (2) | 2.52 (2) | 3.374 (4) | 157 (4) |
| N1B—H1I···S2 | 0.90 (2) | 2.46 (2) | 3.352 (4) | 169 (4) |
| Codes de symétrie : (i) x−1/2, −y+1/2, z+1/2 ; (ii) x, y, z+1 | | | | |
| [Co(SCN)_4_] (C_5_ H_11_ N_3_)_3_, 2Cl | | | | |
| N1—H1N···S1 | 0.89 (1) | 2.48 (2) | 3.340 (3) | 164 (3) |
| N2—H2N···Cl1^ii^ | 0.86 (4) | 2.37 (4) | 3.218 (3) | 168 (3) |
| N3—H3A···Cl1^iii^ | 0.91 (2) | 2.32 (2) | 3.220 (3) | 172 (3) |
| N3—H3B···Cl1^iv^ | 0.92 (2) | 2.30 (2) | 3.217 (3) | 171 (3) |
| N3—H3C···Cl1 | 0.91 (2) | 2.39 (2) | 3.283 (3) | 166 (3) |
| Symmetry codes: (ii) x, y, z+1; (iii) −x+2, −y+1, −z+1; (iv) −x+1, −y+1, −z+1. | | | | |
